# Correction: SEC14-like condensate phase transitions at plasma membranes regulate root growth in *Arabidopsis*

**DOI:** 10.1371/journal.pbio.3003340

**Published:** 2025-08-12

**Authors:** Chen Liu, Andriani Mentzelopoulou, Fotini Papagavriil, Prashanth Ramachandran, Artemis Perraki, Lucas Claus, Sebastian Barg, Peter Dörmann, Yvon Jaillais, Philipp Johnen, Eugenia Russinova, Electra Gizeli, Gabriel Schaaf, Panagiotis Nikolaou Moschou

There is an error in affiliation 6 for authors Lucas Claus and Eugenia Russinova. The correct affiliation 6 is: Center for Plant Systems Biology, VIB, Ghent, Belgium.

## References

[1] Liu C, Mentzelopoulou A, Papagavriil F, Ramachandran P, Perraki A, Claus L, et al. SEC14-like condensate phase transitions at plasma membranes regulate root growth in *Arabidopsis*. PLoS Biol. 2023;21(9):e3002305. doi: 10.1371/journal.pbio.3002305 37721949 PMC10538751

