# Supplementary material for: SEC14-like condensate phase transitions at plasma membranes regulate root growth in Arabidopsis
Source: PLoS Biol. 2023 Sep 18;21(9):e3002305. doi: 10.1371/journal.pbio.3002305 (PMC10538751; doi:10.1371/journal.pbio.3002305)
Supplement: S1 File — (PDF) [file pbio.3002305.s006.pdf]

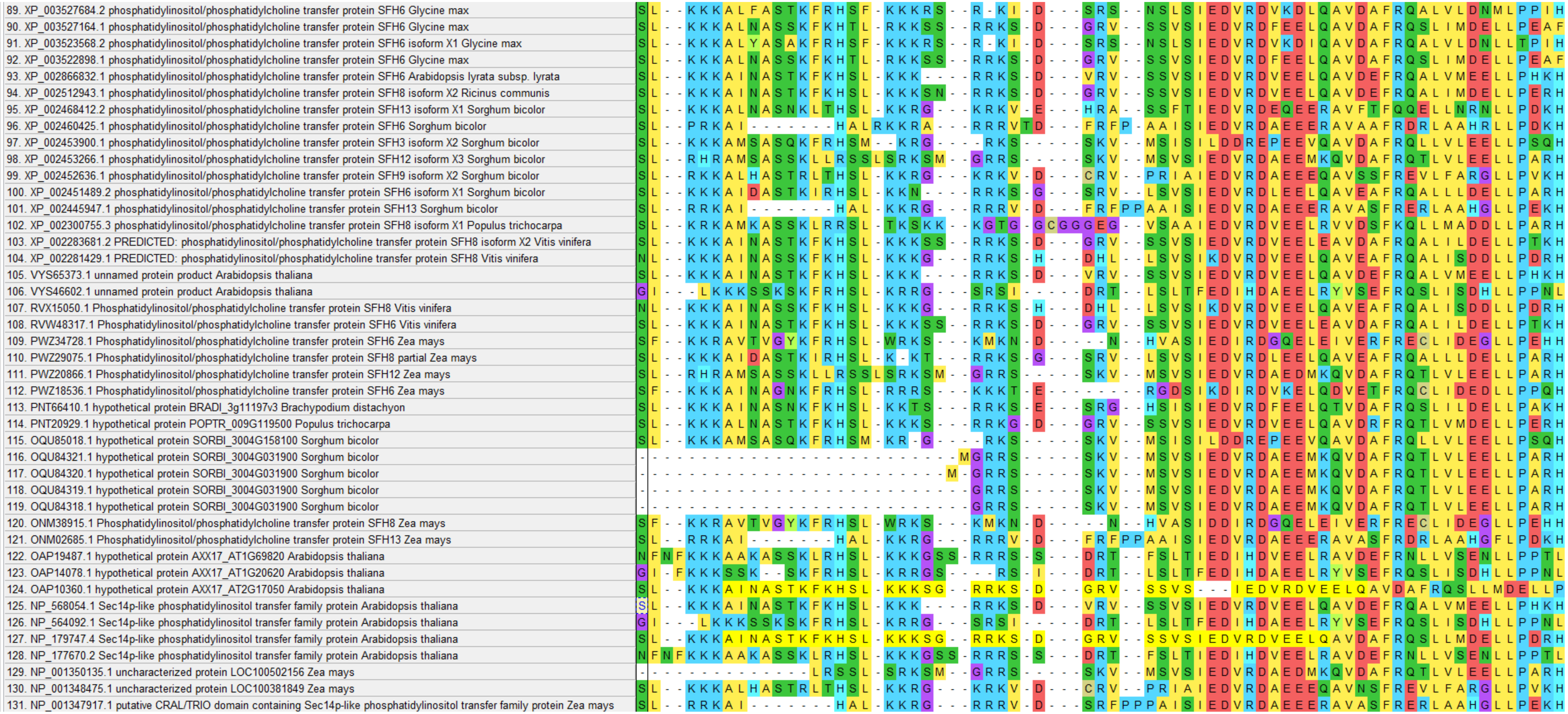
89. XP_003527684.2 phosphatidylinositol/phosphatidylcholine transfer protein SFH6 Glycine max
90. XP_003527164.1 phosphatidylinositol/phosphatidylcholine transfer protein SFH6 Glycine max
91. XP_003523568.2 phosphatidylinositol/phosphatidylcholine transfer protein SFH6 isoform X1 Glycine max
92. XP_003522898.1 phosphatidylinositol/phosphatidylcholine transfer protein SFH6 Glycine max
93. XP_002866832.1 phosphatidylinositol/phosphatidylcholine transfer protein SFH6 Arabidopsis lyrata subsp. lyrata
94. XP_002512943.1 phosphatidylinositol/phosphatidylcholine transfer protein SFH8 isoform X2 Ricinus communis
95. XP_002468412.2 phosphatidylinositol/phosphatidylcholine transfer protein SFH13 isoform X1 Sorghum bicolor
96. XP_002460425.1 phosphatidylinositol/phosphatidylcholine transfer protein SFH6 Sorghum bicolor
97. XP_002453900.1 phosphatidylinositol/phosphatidylcholine transfer protein SFH3 isoform X2 Sorghum bicolor
98. XP_002453266.1 phosphatidylinositol/phosphatidylcholine transfer protein SFH12 isoform X3 Sorghum bicolor
99. XP_002452636.1 phosphatidylinositol/phosphatidylcholine transfer protein SFH9 isoform X2 Sorghum bicolor
100. XP_002451489.2 phosphatidylinositol/phosphatidylcholine transfer protein SFH6 isoform X1 Sorghum bicolor
101. XP_002445947.1 phosphatidylinositol/phosphatidylcholine transfer protein SFH13 Sorghum bicolor
102. XP_002300755.3 phosphatidylinositol/phosphatidylcholine transfer protein SFH8 isoform X1 Populus trichocarpa
103. XP_002283681.2 PREDICTED: phosphatidylinositol/phosphatidylcholine transfer protein SFH8 isoform X2 Vitis vinifera
104. XP_002281429.1 PREDICTED: phosphatidylinositol/phosphatidylcholine transfer protein SFH8 Vitis vinifera
105. VYS65373.1 unnamed protein product Arabidopsis thaliana
106. VYS46602.1 unnamed protein product Arabidopsis thaliana
107. RVX15050.1 Phosphatidylinositol/phosphatidylcholine transfer protein SFH8 Vitis vinifera
108. RVW48317.1 Phosphatidylinositol/phosphatidylcholine transfer protein SFH6 Vitis vinifera
109. PWZ34728.1 Phosphatidylinositol/phosphatidylcholine transfer protein SFH6 Zea mays
110. PWZ29075.1 Phosphatidylinositol/phosphatidylcholine transfer protein SFH8 partial Zea mays
111. PWZ20866.1 Phosphatidylinositol/phosphatidylcholine transfer protein SFH12 Zea mays
112. PWZ18536.1 Phosphatidylinositol/phosphatidylcholine transfer protein SFH6 Zea mays
113. PNT66410.1 hypothetical protein BRADI_3g11197v3 Brachypodium distachyon
114. PNT20929.1 hypothetical protein POPTR_009G119500 Populus trichocarpa
115. OQU85018.1 hypothetical protein SORBI_3004G158100 Sorghum bicolor
116. OQU84321.1 hypothetical protein SORBI_3004G031900 Sorghum bicolor
117. OQU84320.1 hypothetical protein SORBI_3004G031900 Sorghum bicolor
118. OQU84319.1 hypothetical protein SORBI_3004G031900 Sorghum bicolor
119. OQU84318.1 hypothetical protein SORBI_3004G031900 Sorghum bicolor
120. ONM38915.1 Phosphatidylinositol/phosphatidylcholine transfer protein SFH8 Zea mays
121. ONM02685.1 Phosphatidylinositol/phosphatidylcholine transfer protein SFH13 Zea mays
122. OAP19487.1 hypothetical protein AXX17_AT1G69820 Arabidopsis thaliana
123. OAP14078.1 hypothetical protein AXX17_AT1G20620 Arabidopsis thaliana
124. OAP10360.1 hypothetical protein AXX17_AT2G17050 Arabidopsis thaliana
125. NP_568054.1 Sec14p-like phosphatidylinositol transfer family protein Arabidopsis thaliana
126. NP_564092.1 Sec14p-like phosphatidylinositol transfer family protein Arabidopsis thaliana
127. NP_179747.4 Sec14p-like phosphatidylinositol transfer family protein Arabidopsis thaliana
128. NP_177670.2 Sec14p-like phosphatidylinositol transfer family protein Arabidopsis thaliana
129. NP_001350135.1 uncharacterized protein LOC100502156 Zea mays
130. NP_001348475.1 uncharacterized protein LOC100381849 Zea mays
131. NP_001347917.1 putative CRAL/TRIO domain containing Sec14p-like phosphatidylinositol transfer family protein Zea mays
