# Supplementary figures and images for: SEC14-like condensate phase transitions at plasma membranes regulate root growth in Arabidopsis
Source: PLoS Biol. 2023 Sep 18;21(9):e3002305. doi: 10.1371/journal.pbio.3002305 (PMC10538751; doi:10.1371/journal.pbio.3002305)

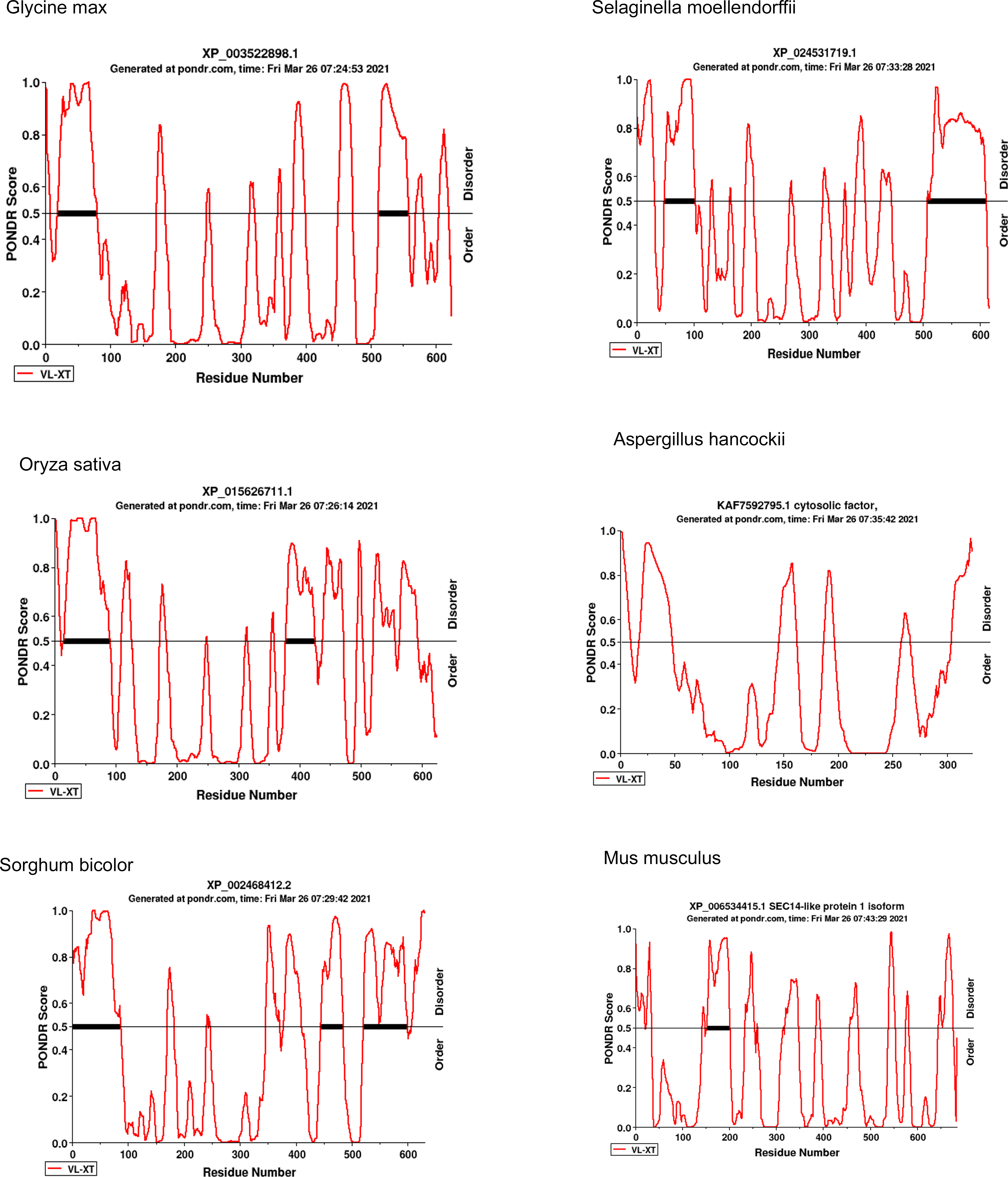

Supplement: S2 File — (TIF) [file pbio.3002305.s007.tif]

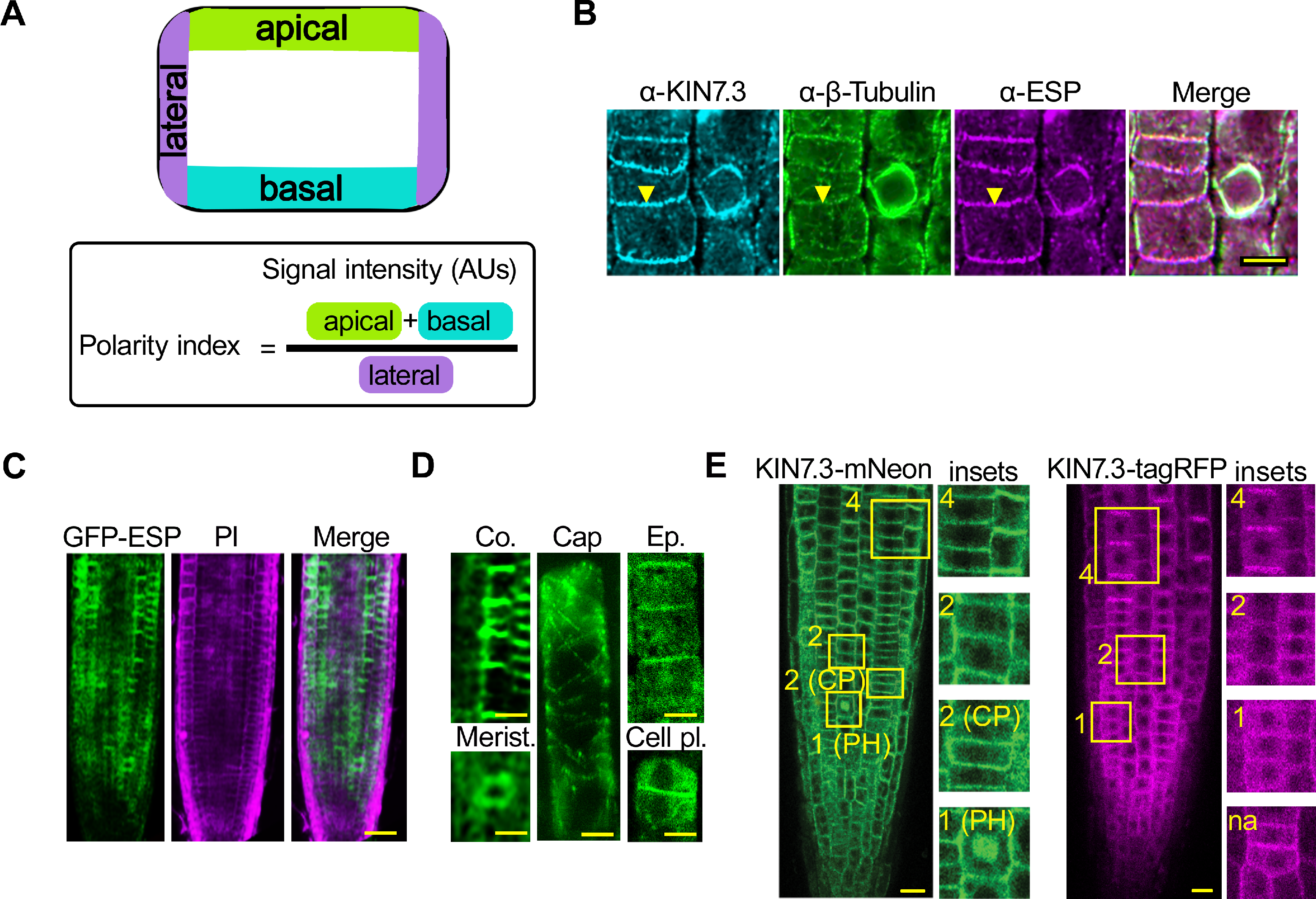

Supplement: S1 Fig — (A) Apical/basal/lateral PM domain nomenclature and polarity index determination. (B) Micrographs of α-ESP/α-KIN7.3 localizations at similar polar domains (counterstained with α-β-tubulin; region 4, experiment replicated 4 times). Scale bars, 5 μm. (C) Micrograph of the ESP-expressing estradiol inducible line (KIN7.3pro>XVE>GFP-ESP; 5 DAG; 20 μM estradiol, 16 h). KIN7.3 promoter was used, as the ESP promoter was not functional. Scale bars, 5 μm. ESP showed polarization only in the distal meristematic region, while in the proximal meristem region, ESP was apolar. Micrographs were obtained in low resolution (512 × 512 with minimal exposure and no averaging), due to photobleaching and low expression levels of ESP (experiment replicated more than 10 times with variable expression). Scale bar, 50 μm. (D) Digitally zoomed-in confocal micrographs (deconvoluted, 5 DAG) from root tip cells of the KIN7.3pro>XVEpro>GFP-ESP. Merist., proximal meristematic cell (note the diffused cytoplasmic signal). Ep., epidermis; Co., cortex; Cell pl., cell plate; Cap, lateral root cap. Note the MT-binding of ESP in the lateral root cap cell where MTs are highly bundled (as also described in [18,28]). (E) Micrographs of roots expressing RPS5apro:KIN7.3-mNeon in the k135 background at the indicated regions (5 DAG). Right: micrographs from lines expressing RPS5apro:KIN7.3-tagRFP in the k135 background (5 DAG). CP, cell plate; PH, phragmoplast; na, not taken from the micrograph on the left. The experiment was replicated 4 times. Scale bars, 20 μm. Raw data can be found in the Supporting information section (S1 Data). DAG, day after germination; Ep., epidermis; ESP, EXTRA SPINDLE POLES; KISC, kinesin-separase complex; MT, microtubule; PM, plasma membrane. (TIF) [file pbio.3002305.s012.tif]

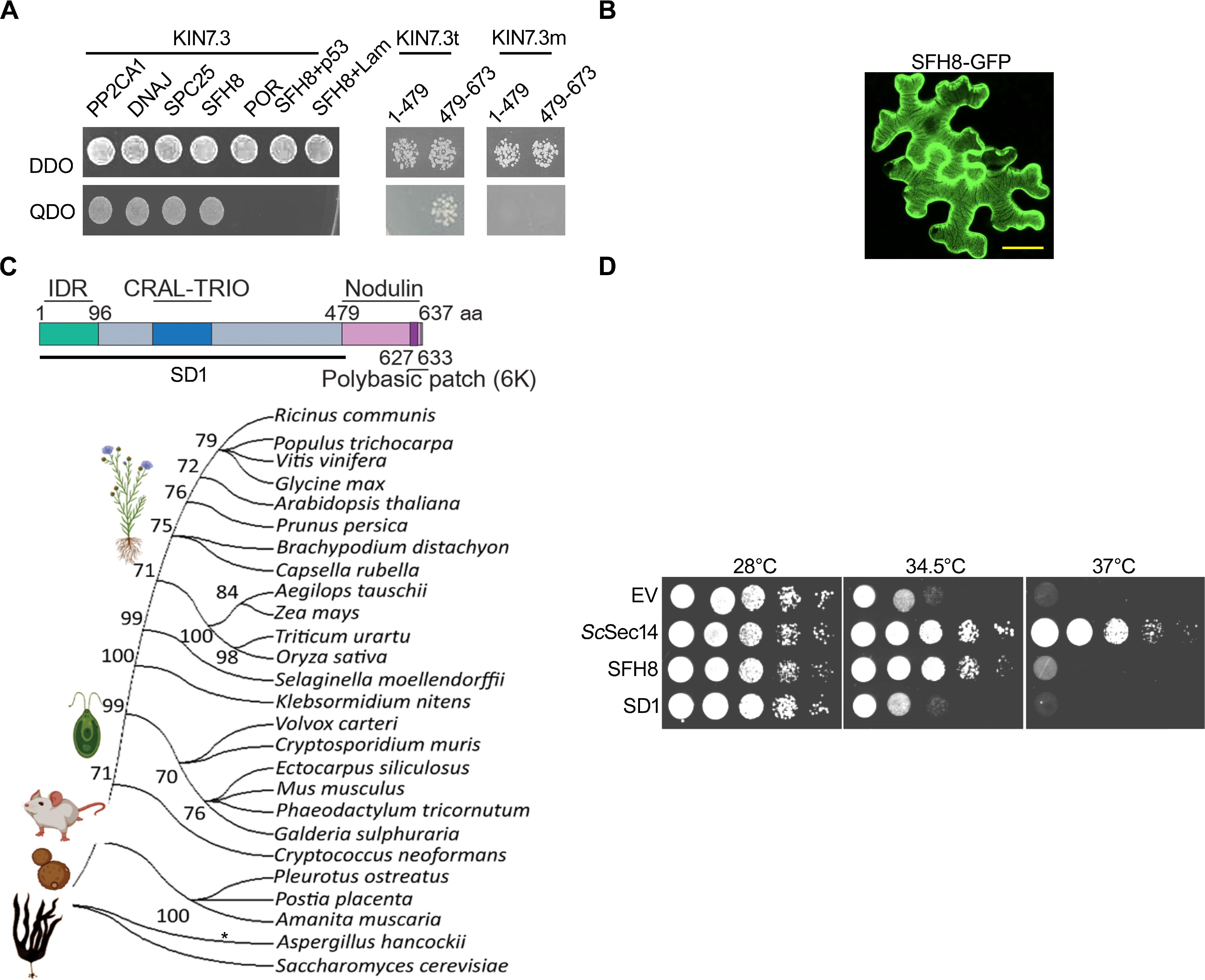

Supplement: S2 Fig — (A) Y2H between BD fusion with KIN7.3 (bait) and 5 putative interactors (AT2G21520 (SFH8) PP2CA1, DNAJ, SPC25, and PORcino fused to activation domain (pray); see also S1 Text for a description of the KIN7.3 interactors, which were not followed herein). DDO, double dropout (control); QDO, quadruple dropout (selection of interactions). Negative controls: p53 and lamin (Lam). Right: Kin7.3 tail (t, C terminus) or Kin7.3 motor (m, N terminus) interaction with SFH8 lacking the nodulin motif (1–479) or with the nodulin motif (479–673). For the architecture of SFH8, see (C). The experiment was replicated 5 times. (B) Micrograph (maximum intensity projection) showing SFH8-GFP localization in N. benthamiana leaf epidermis (3 days post infiltration and driven by the 35Spro). Scale bar, 20 μm. The experiment was replicated 20 times. (C) SFH8 architecture; numbers indicate aa truncations used throughout the paper. SD1 corresponds to amino acid residues 1–478. IDR, intrinsically disordered region (1–96 aa). The CRAL-TRIO domain binds small lipophilic molecules and is named after the cellular retinaldehyde-binding protein and TRIO guanine exchange factor. Lower: phylogenetic analysis of SFH8. Numbers on branches indicate bootstrap values with a confidence cutoff of 70. (D) Budding yeast temperature-sensitive sec14-1ts loss-of-function mutant complementation by full-length SFH8 or SD1 (1–479; see (B)). At 37°C, the complementation was moderate, likely due to the instability of the protein at elevated temperatures (physiological Arabidopsis growth temperature which is between 22–28°C). Dilution series: 0 (undiluted)–10−4. The experiment was replicated 3 times. Raw data can be found in the Supporting information section (S1 Data). BD, binding domain; KISC, kinesin-separase complex; SFH8, SEC FOURTEEN-HOMOLOG8; Y2H, yeast two-hybrid. (TIF) [file pbio.3002305.s013.tif]

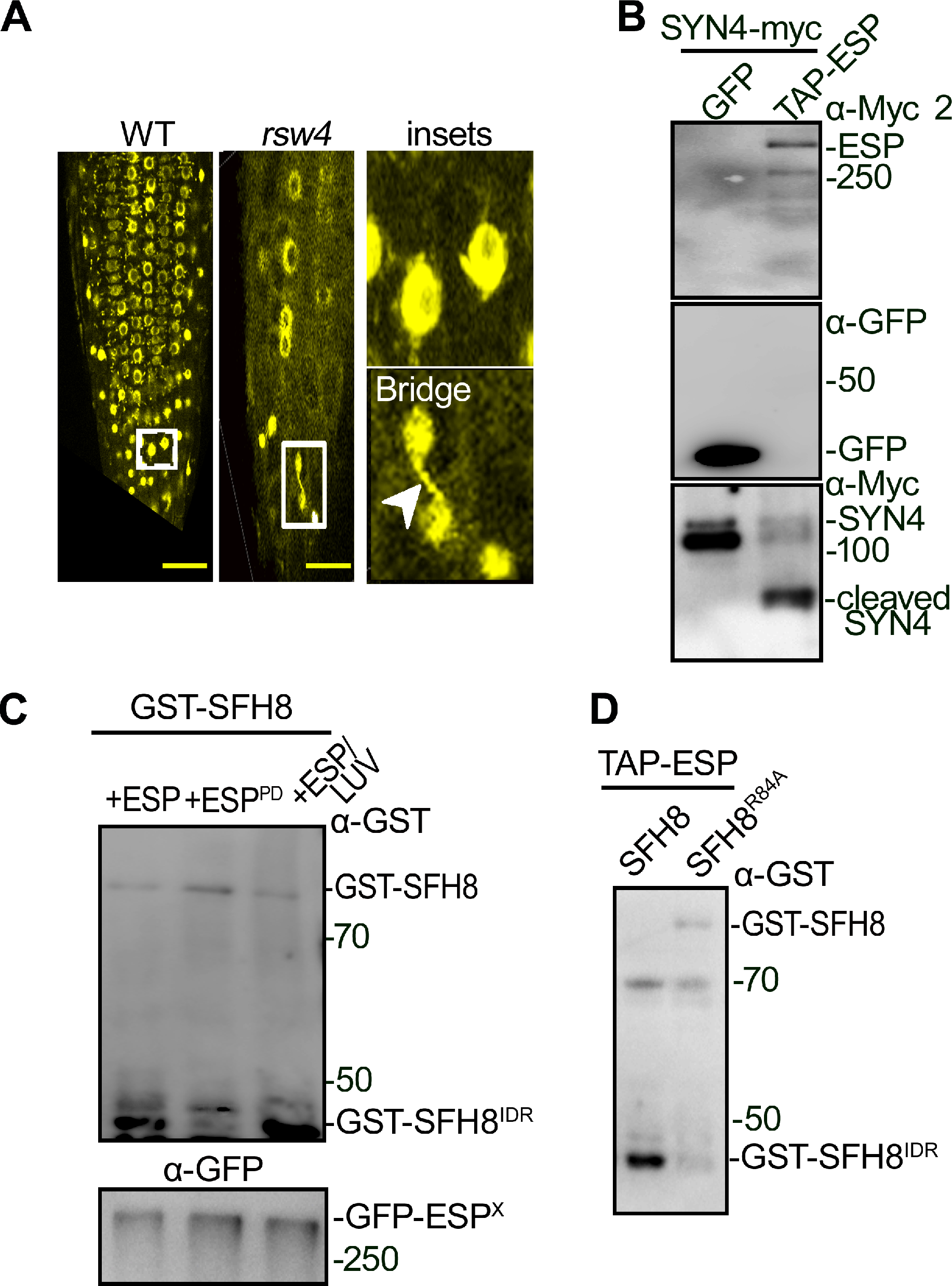

Supplement: S3 Fig — (A) Establishment of a positive cleavage control for ESP: the mitotic cohesin SYN4 is an in vivo ESP target (see also relevant S1 Text on the use of SYN4). Micrographs of epidermal root cells expressing SYN4pro:CFP-SYN4-YFP in WT or the temperature-sensitive ESP mutant rsw4 (at the restrictive temperature of 28°C for 72 h, 5–7 DAG). Insets show chromosomal bridges in rsw4 due to the presumptive lack of the mitotic SYN4 kleisin subunit cleavage by ESP (epidermis); the white arrowhead denotes a chromosomal bridge. The experiment was replicated 5 times (with 1–3 bridges evident per root). Scale bars, 40 μm. (B) Immunoblots showing the in vitro cleavage of SYN4 on beads purified from leaves of N. benthamiana (using myc, 35Spro:SYN4-myc), in the presence of immunopurified from N. benthamiana aTAP-ESP (or GFP-aTAP) preactivated by CyclinD (Materials and methods for details on purification). Incubation was for 1 h at 37°C. The experiment was replicated 3 times. (C) In vitro GST-SFH8 cleavage by immunopurified from N. benthamiana GFP-tagged variants of ESP, ESPPD (protease dead [81], and ESP in the presence of LUVs (made from phosphatidylcholine and containing 10 mol %phospatidylserine). The beads carrying GFP-tagged ESP variants in the ±LUVs were coincubated with GST-SFH8 at 37°C for 1 h. Note that proteolytically inactive ESP failed to cleave SFH8, while cleavage is sustained in the presence of liposomes (ESP/LUV). The produced GST-SFH8IDR is shown (band below 50 kDa). As the observed compromised full-length SFH8 transfer from the SDS-PAGE to the membrane, the immunoreactive signal increment of the SFH8IDR/SFH8 ratio upon cleavage was not proportional to the full-length SFH8 depletion. Note that the band approximately 50 kDa was detected in all samples and thus could correspond to a nonspecific cleavage product. Detection of GFP-ESP (WT or PD) is shown at the bottom. The experiment was replicated 4 times. (D) GST-SFH8R84A is not cleaved by aTAP-ESP (similar experimental s [file pbio.3002305.s014.tif]

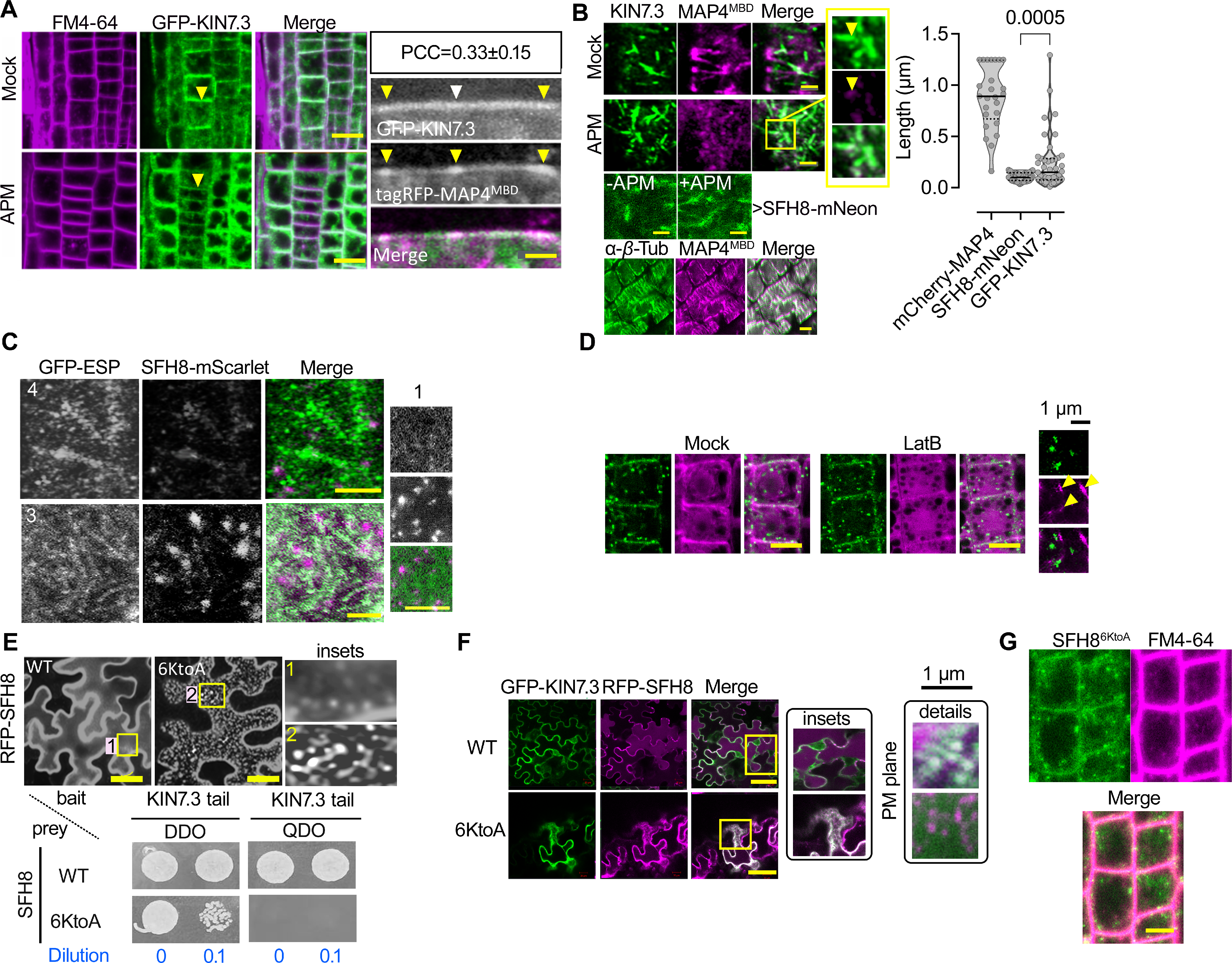

Supplement: S4 Fig — (A) Micrographs of KIN7.3pro:GFP-KIN7.3/35Spro:tagRFP-MAP4MBD expressing lines (5 DAG, epidermis region 3) counterstained with FM4-64, ±APM (10 nM, 1 h). Micrographs are representative of an experiment replicated twice. Scale bars, 12 μm. Right: micrographs of lines coexpressing KIN7.3pro:GFP-KIN7.3/35Spro:tagRFP-MAP4MBD at cell contours (5 DAG, epidermis region 3). The box at the top right shows PCC calculated at the regions indicated with the arrowheads, which, in the case of MAP4MBD, denote PM-attached MT bundles (data are means ± SD, N = 3, n = 5–10 cells per experiment). The white arrowhead denotes a slightly increased colocalization between GFP-KIN7.3/tagRFP-MAP4MBD. Scale bar, 1 μm. (B) Micrograph showing the partial colocalization of GFP-KIN7.3 filaments with MAP4MBD (in root region 3) ±APM (10 nM, 1 h). Note that KIN7.3 filaments can associate with bundled-MT remnants (reminiscent of clusters; insets with arrowheads). Micrographs are representative of an experiment replicated twice. Lower: micrograph showing SFH8 filaments and their resistance to APM (10 nM, 1 h; region 3); colocalization between tagRFP-MAP4MBD/α-β-tubulin signals, confirming that tagRFP-MAP4MBD follows tubulin localization. Note though that overexpression of MAP4 protein induces significant MT bundling. Scale bars, 0.1 μm (for filaments) or 3.4 μm for colocalization (tagRFP-MAP4 MBD/α-β-tubulin). Right: quantification of KIN7.3/SFH8 filaments’ length at the PM (N = 6 pooled experiments, n = 5–9 cells measuring 10 filaments in each; p-value was calculated by Wilcoxon). GFP-KIN7.3 showed more variance, indicative of KIN7.3 association with MT filaments/bundles, as well. Scale bars, 1 μm. (C) Micrographs of the line coexpressing KIN7.3pro>XVEpro>GFP-ESP/RPS5apro:mScarlet-SFH8, after estradiol induction (20 μM, 16–24 h; 5 DAG, regions indicated on micrographs-epidermis, cell surface). Images were obtained at maximum scanning speed (40 frames per second) using super-resolution. The experiment w [file pbio.3002305.s015.tif]

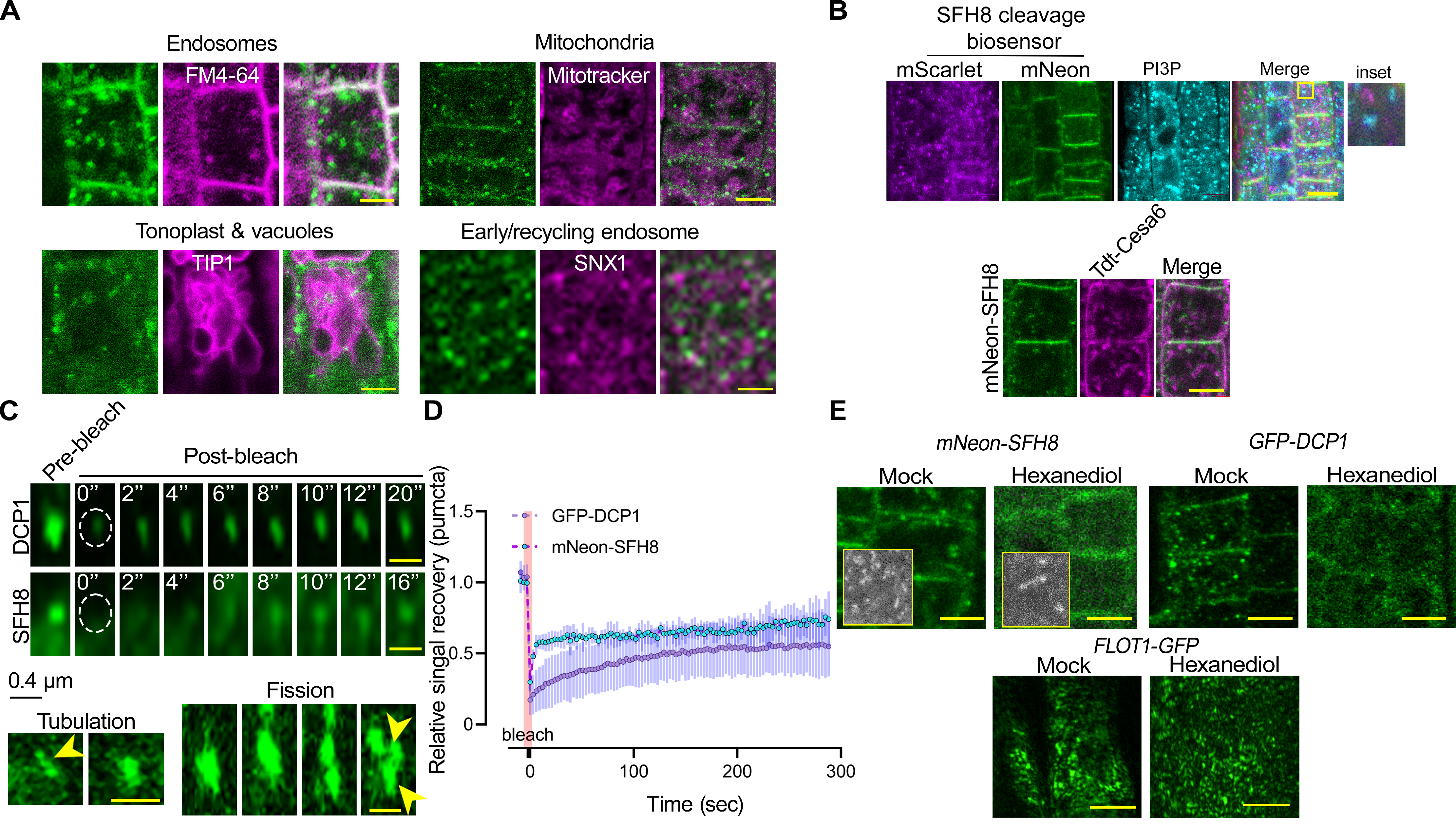

Supplement: S5 Fig — (A) Micrographs from RPS5apro:mNeon-SFH8 expressing lines counterstained with FM4-64 (endosomes and PM) or mitotracker (mitochondria; upper panel); lines coexpressing RPS5apro:mNeon-SFH8 with 35Spro:TIP1-RFP (tonoplast intrinsic protein 1), or 35Spro:SNX1-RFP (sorting nexin 1; TGN; lower panel). In some cases, the duration of tracking was limited to the amount of time that particles remained in a single focal plane, as the required acquisition rate did not permit the collection of z-stacks (voxels); the median track length was 35 frames, corresponding to 20 s of imaging. PCC analyses failed to show colocalization of SFH8 cytoplasmic puncta with any of the markers/dyes used. The experiment was replicated 3 times. Scale bars, 2 μm. (B) SFH8 does not colocalize with PtdIns(3)P-positive structures in vivo (endosomes and autophagosomes). Micrograph of lines coexpressing mScarlet-SFH8-mNeon (green; RPS5apro:6xhis-3xFLAG(HF)-mScarlet-SFH8-mNeon; denoted as “cleavage biosensor”; see also Fig 3) with 1xPXp40 (tagged with CFP and expressed under pUBI10). Inset (right) shows a lack of colocalization between PtdIns(3)P and mScarlet-SFH8IDR. Scale bar, 4 μm. Right: micrograph of lines coexpressing RPS5apro:mNeon-SFH8 with Tdt-CESA6 (CELLULOSE SYNTHASE 6 under the 35Spro). The experiment was replicated 3 times for various developmental stages (2–7 DAG), and root tissues (regions 1–4). Scale bar, 4 μm. (C) Upper: FRAP signal recovery as a fraction of time in lines expressing 35Spro:GFP-DCP1 (forming cytoplasmic condensates known as processing bodies [82]) or SFH8pro:mNeon-SFH8. The ROIs were set on mobile cytoplasmic puncta (at the midsection; 7 DAG, epidermis regions 3–4). The experiment was replicated 3 times. Scale bars, 0.4 μm. Lower: time-lapse imaging (2-s time interval) of mNeon-SFH8 puncta. Arrowheads denote the tubulating (left) or coalescing puncta (right). (D) FRAP signal recovery as a fraction of time, of DCP1 and SFH8 (relevant to C). The red faded band parallel to th [file pbio.3002305.s016.tif]

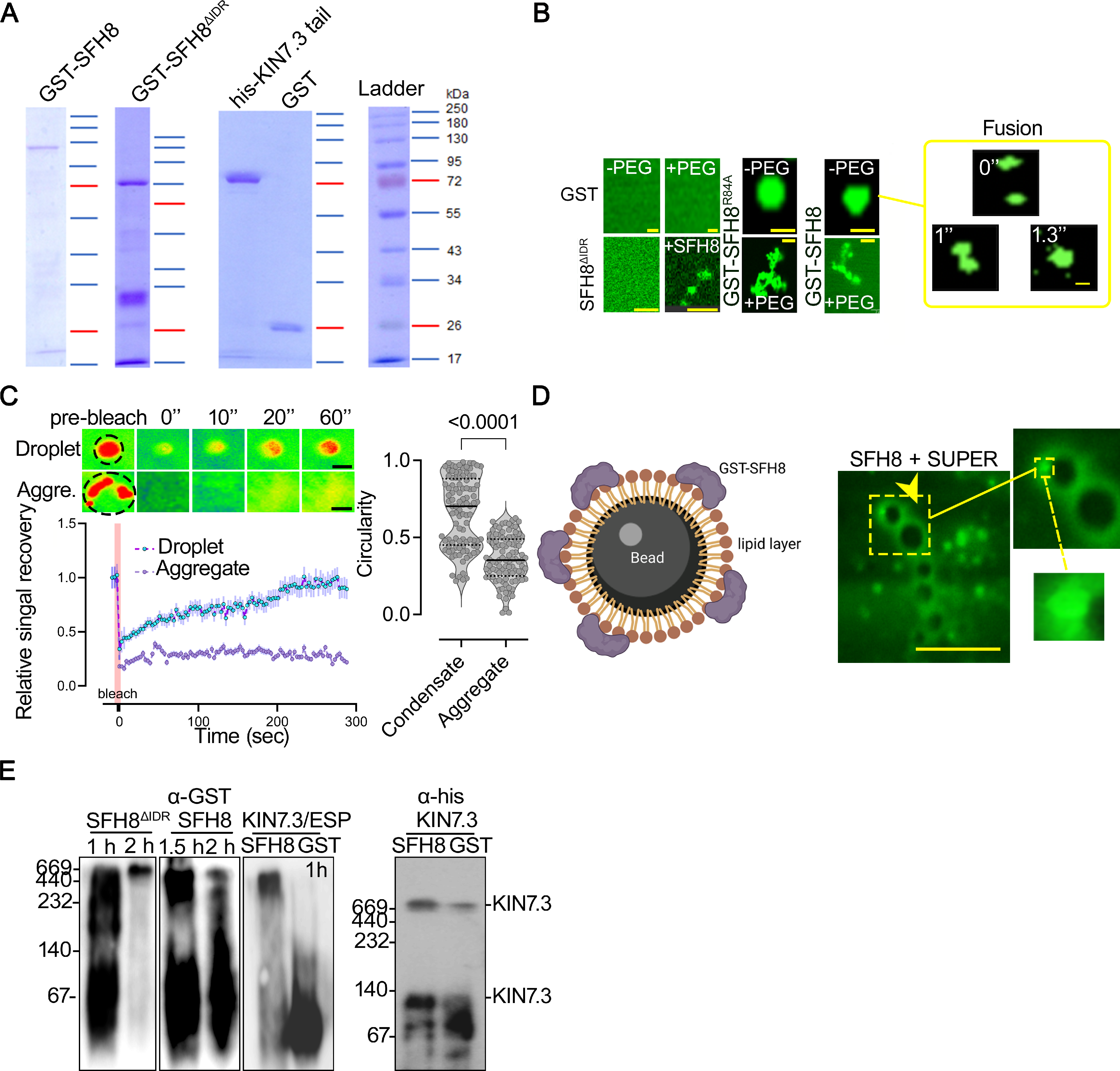

Supplement: S6 Fig — (A) Examples of purified recombinant proteins from E. coli for assays used below visualized on SDS-PAGE (10%). We note that variations in each purification were observed. (B) Micrographs of in vitro recombinant GST, GST-SFH8, GST-SFH8ΔIDR, and GST-SFH8R84A proteins stained with Alexa638 in the presence or absence of the crowding agent PEG3000 in LLPS conditions. Note that in the presence of PEG3000, SFH8 and SFH8R84A switch to agglomerate-like states, while coincubation of GST-SFH8ΔIDR with GST-SFH8 induced the filamentous transition of both proteins. The experiment was replicated 3 times. Right detail (fusion): GST-SFH8 droplet fusion in the same experiment. (C) Representative confocal high-speed micrographs showing droplet and aggregate signal recovery in FRAP. Scale bars, 0.2 μm. The experiment was replicated 3 times. Lower: FRAP signal recovery as a fraction of time of GST-SFH8R84A droplets and aggregates. The red faded band parallel to the y-axis indicates laser iteration time. A quantification of the circularity of condensates or aggregates is also shown (lower right). Data are means ± SD (N = 5 pooled experiments; n = 1 assay). (D) Super-resolution micrographs from a SUPER template experiment show that GST-SFH8 labeled with Alexa638 (0.1 μM of GST-SFH8 in the assay) can form liquid-like droplets on membranes. Furthermore, GST-SFH8 showed an increased propensity to undergo LLPS in the presence of SUPER templates (see arrowhead denoting droplet; some droplets were released in the bulk phase). The experiment was replicated 3 times. Scale bar, 20 μm. (E) Native gel electrophoresis and detection by α-GST of SFH8ΔIDR and full-length SFH8 showing the time-depended conversion of the 2 proteins to high molecular weight assemblies. Very few agglomerations were observed for GST-SFH8. Note that in the presence of Kin7.3/ESP GST-SFH8 converted faster to high-molecular weight assemblies (GST: negative control). Right immunoblot: detection of Kin7.3 using α-his. Immunoblots [file pbio.3002305.s017.tif]

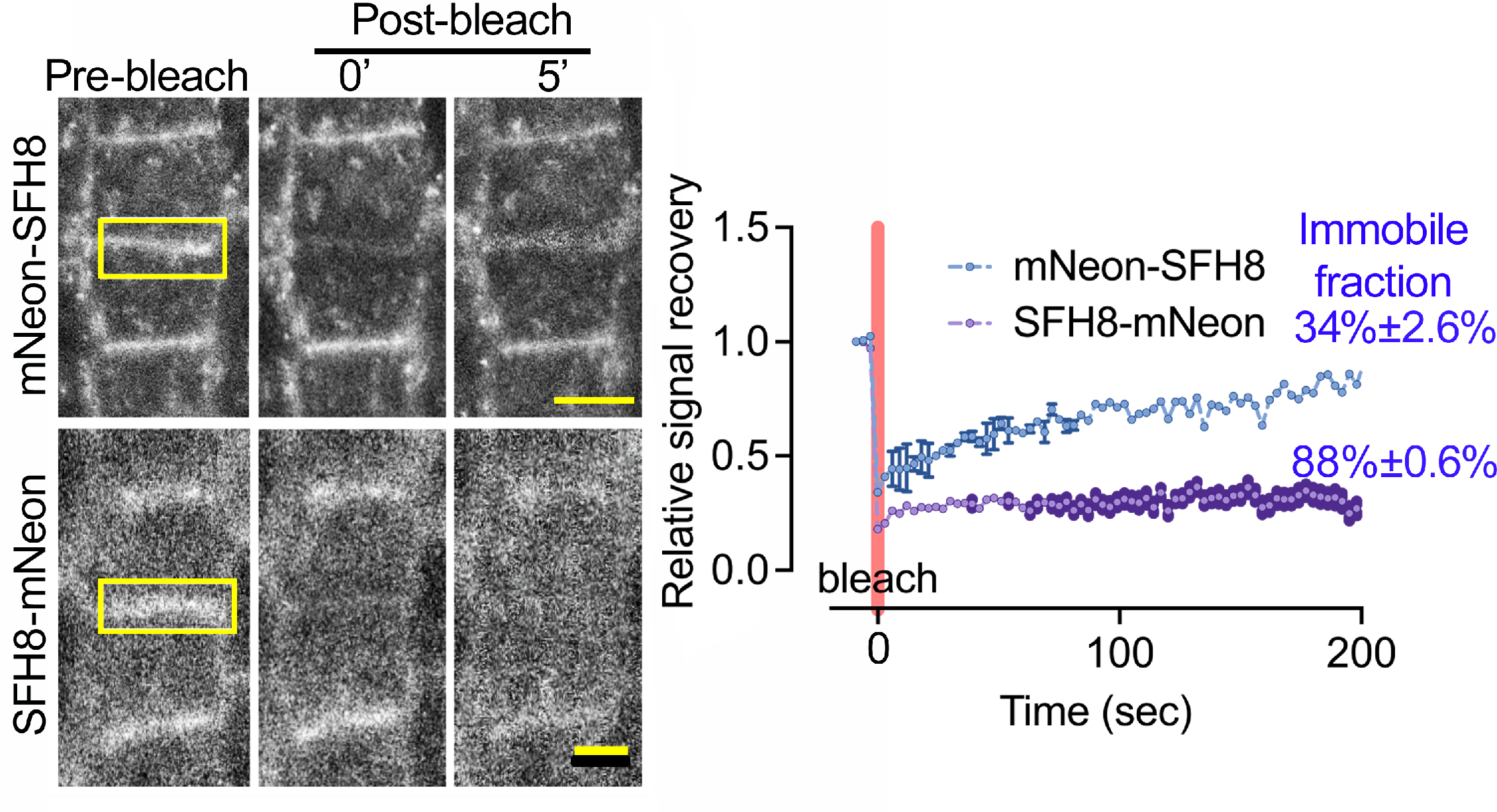

Supplement: S7 Fig — Microgrpahs showing FRAP signal recovery of the N or C-terminally tagged SFH8 with mNeon on the PM (SFH8pro in sfh8 background, 5–7 DAG, epidermis region 3), implying increased diffusion (due to cleavage, liquidity, and dwelling time of clusters). The yellow rectangular denotes the ROI that was bleached. The experiment was replicated 3 times. Scale bars, 5 μm. Right: corresponding FRAP signal recovery as a fraction of time. Percentages indicate immobile fractions for N- or C-terminally tagged SFH8. The red faded band parallel to the y-axis indicates laser iteration time (bleach). Data are means ± SD (N = 3 pooled experiments, n = 1 assay). Raw data can be found in the Supporting information section (S1 Data and S1 Raw Images). DAG, day after germination; FRAP, fluorescence recovery after photobleaching; PM, plasma membrane; ROI, region of interest; SFH8, SEC FOURTEEN-HOMOLOG8. (TIF) [file pbio.3002305.s018.tif]

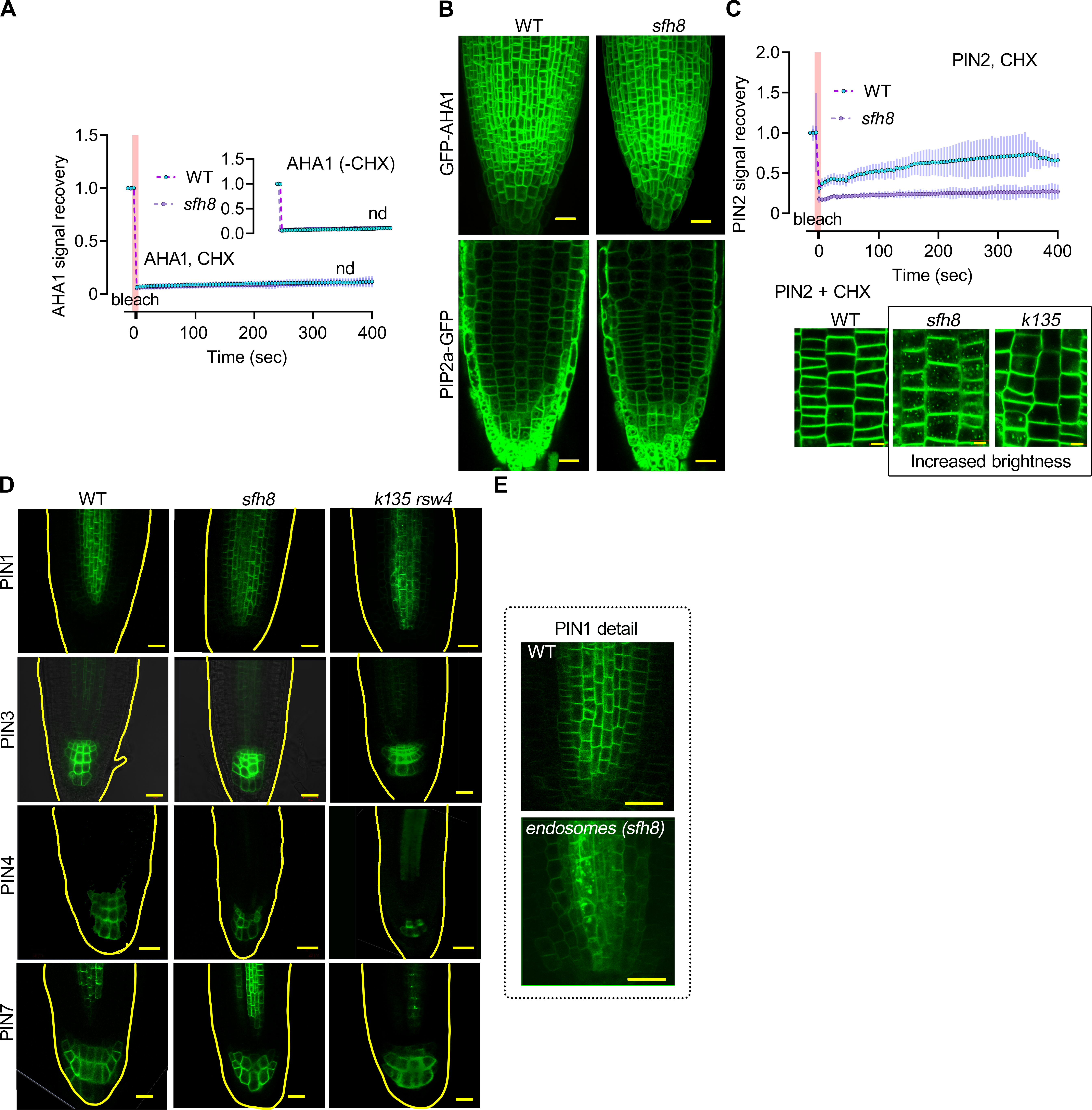

Supplement: S8 Fig — (A) FRAP signal recovery as a fraction of time from lines expressing 35Spro:GFP-AHA1 in WT or sfh8 (upper; inset graph without CHX). The red faded band parallel to the y-axis indicates laser iteration time (bleach). Data are means ± SD (N = 3 pooled experiments, n = 3 assays). nd, no difference. (B) Micrographs of lines expressing 35Spro:GFP-AHA1, or 35Spro:PIP2a-GFP in WT or sfh8 (5 DAG, epidermis and cortex). The experiment was replicated twice. Scale bars, 20 μm. (C) FRAP signal recovery as a fraction of time from lines expressing PIN2-GFP in WT or sfh8 in the presence of CHX. The red faded band parallel to the y-axis indicates laser iteration time (bleach). Data are means ± SD (N = 3 pooled experiments, n = 5–8 assays). Lower: PIN2-GFP retention in sfh8 and k135 endosomes (30 min CHX treatment; 5 DAG, epidermis and cortex region 3). Note that CHX did not lead to the dissolution of PIN2 endosomes. To normalize PIN2 levels and visualize endosomes in mutants, brightness was increased by 50%. The experiment was replicated twice. Scale bars, 3 μm. (D) Micrographs showing the localization of C-terminally tagged PINs at polar domains expressed in WT, sfh8, and rsw4 under native promoters (24 h at the restrictive temperature 28°C; 5 DAG). Right: localization details for PIN1; note that in some cases, PIN1-GFP accumulated in endosomes in the sfh8 and k135 rsw4, suggesting that PIN1 delivery was also compromised albeit to a lesser extent than PIN2 (as also discussed in [28] for the rsw4 mutant). The experiment was replicated 10 times. Scale bars, 20 μm. Note that slight level perturbations at the PM were also observed for PIN3, 4, and 7 in the sfh8 and k135 rsw4 (24 h at the restrictive temperature 28°C; 5 DAG), but due to different patterning of the columella cells in these mutants, these are hard to follow. Raw data can be found in the Supporting information section (S1 Data). CHX, cycloheximide; DAG, day after germination; FRAP, fluorescence recovery after photobleachi [file pbio.3002305.s019.tif]

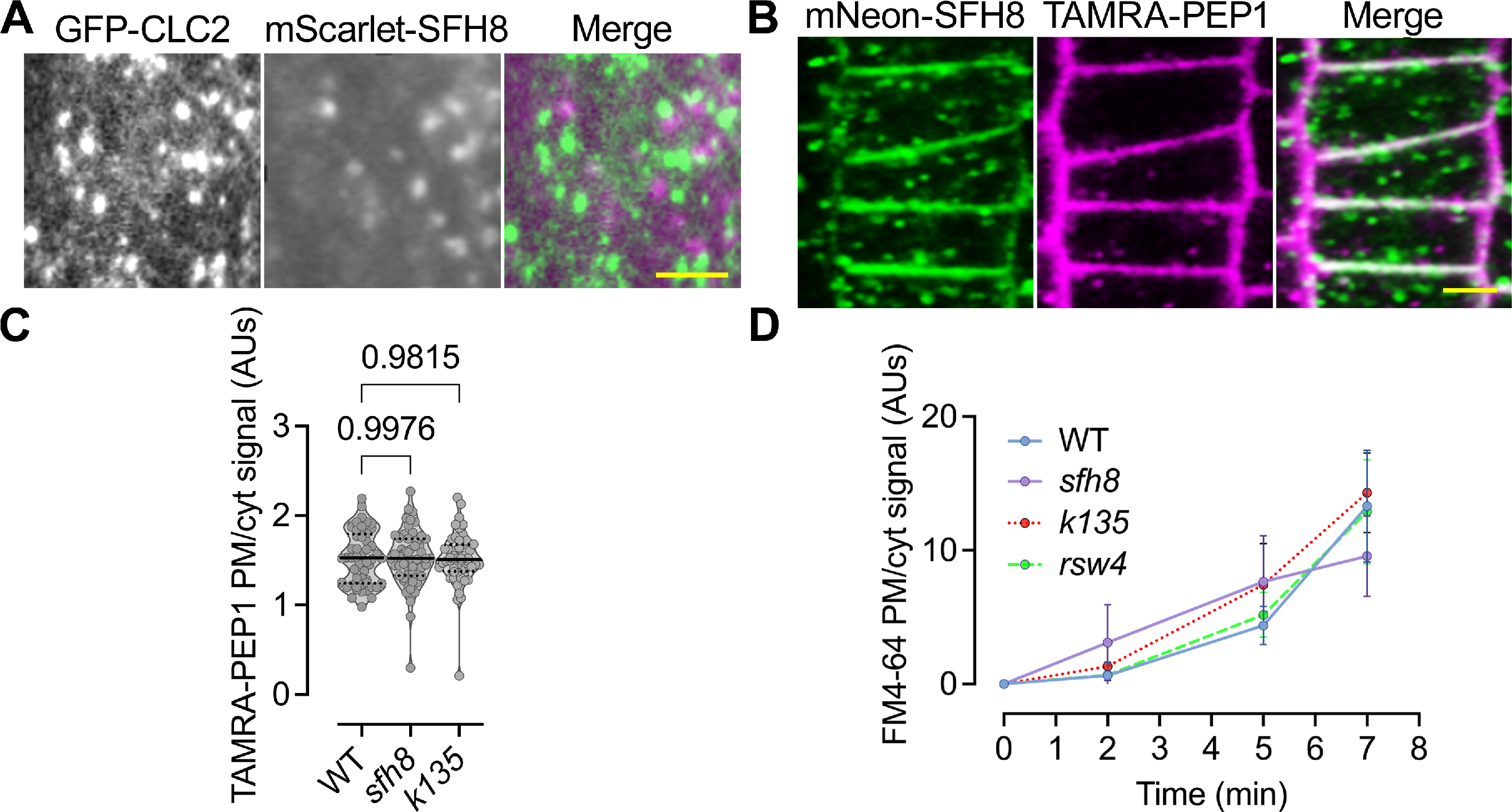

Supplement: S9 Fig — (A) Dual-channel TIRFM showing the lack of colocalization between mScarlet-SFH8 (magenta) and CLC2-GFP (green). The experiment was replicated 5 times. Scale bar, 0.2 μm. (B) Micrographs showing the lack of colocalization between mNeon-SFH8 (SFH8pro, 5 DAG) and TAMRA-PEP1 (left; stains TGN and PM clusters). (C and D) Quantification of TAMRA-PEP1 and FM4-64 uptake in WT, sfh8, and k135 (violin plot and rate plot, respectively; Data are means ± SD, N = 3 pooled experiments, n = 18–25 cells per experiment, p-values were calculated by Wilcoxon). Scale bars, 2 μm. Raw data can be found in the Supporting information section (S1 Data). DAG, day after germination; KISC, kinesin-separase complex; PIN, PINFORMED; PM, plasma membrane; SFH8, SEC FOURTEEN-HOMOLOG8; TGN, trans-Golgi network; TIRFM, total internal reflection fluorescence microscopy; WT, wild type. (TIF) [file pbio.3002305.s020.tif]

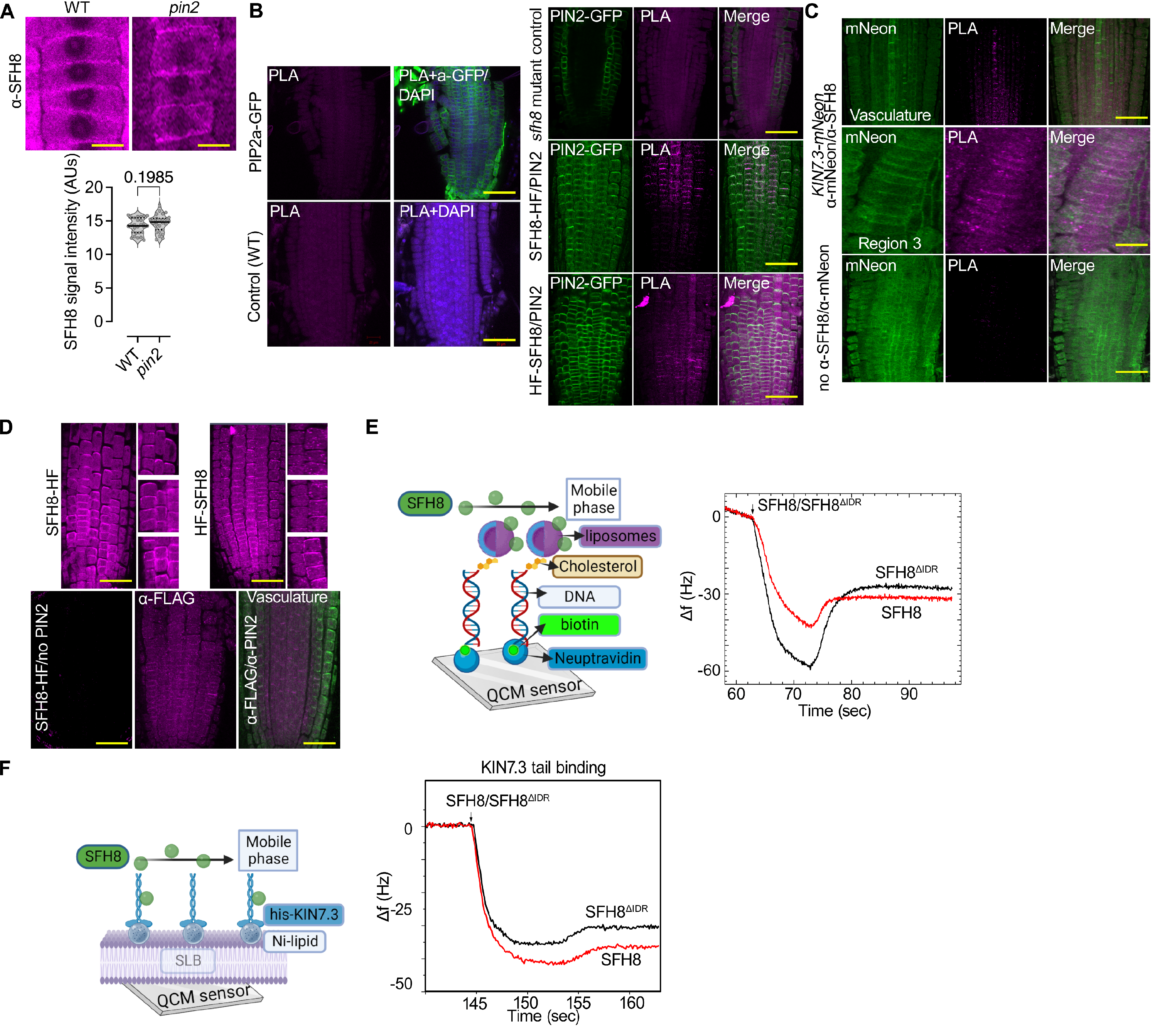

Supplement: S10 Fig — (A) Micrographs showing SFH8 localization (α-SFH8) signal in WT, or pin2 mutant (5 DAG, region 3 cortex), and quantification of SFH8 signal intensity (lower; N = 3 pooled experiments, n = 9 roots with signal calculations from cortex regions 3–4; p-values were calculated by unpaired t test). Scale bars, 5 μm. (B) Technical controls testing the specificity of the PLA approach in our settings (PIN2/SFH8 PLA assay), showing also that detected interactions are selective and SFH8 is not promiscuously interacting with proteins. SFH8 did not interact with PIP2a (α-GFP/α-SFH8), while SFH8-HF (α-FLAG, RPS5apro) interacted extensively with PIN2 at the PM of region 3 onwards (α-GFP; epidermis and cortex). Note the significantly reduced PLA-positive signal in the N-terminally tagged SFH8, HF-SFH8/PIN2 (PLA signal was observed at the cell plate as well). The sfh8 mutant was used as a negative control for the PLA (sometimes a nuclear, likely nonspecific signal was observed in negative controls). The experiments were replicated 5 times. Scale bars, 50 μm. (C) PLA of KIN7.3-mNeon/SFH8 (α-mNeon/α-SFH8). Note the increased interaction towards region 4 (for example, in the vasculature; upper micrographs). The “no -α-SFH8” corresponds to negative control (bottom). The experiment was replicated 5 times. Scale bars, 50 μm (for “region 3” panel, 10 μm). (D) α-FLAG signal in HF-SFH8 (N-terminally tagged) or SFH8-HF (C-terminally tagged) expressing lines (RPS5apro) in sfh8 (5 DAG). Insets denote the localization at the corresponding region. Lower: α-FLAG detection of HF-SFH8 counterstained with α-PIN2 signal (5 DAG). Note the lack of PIN2 from the vasculature and the presence of both SFH8 and PIN2 at the cortex and epidermis. Note also that FLAG-tagged SFH8 localizes like the native SFH8. The experiment was replicated twice. Scale bars, 50 μm. (E) We used the QCM device as a sensitive mass sensor monitoring the frequency response (Δf) of the acoustic wave during binding events on the surface [file pbio.3002305.s021.tif]

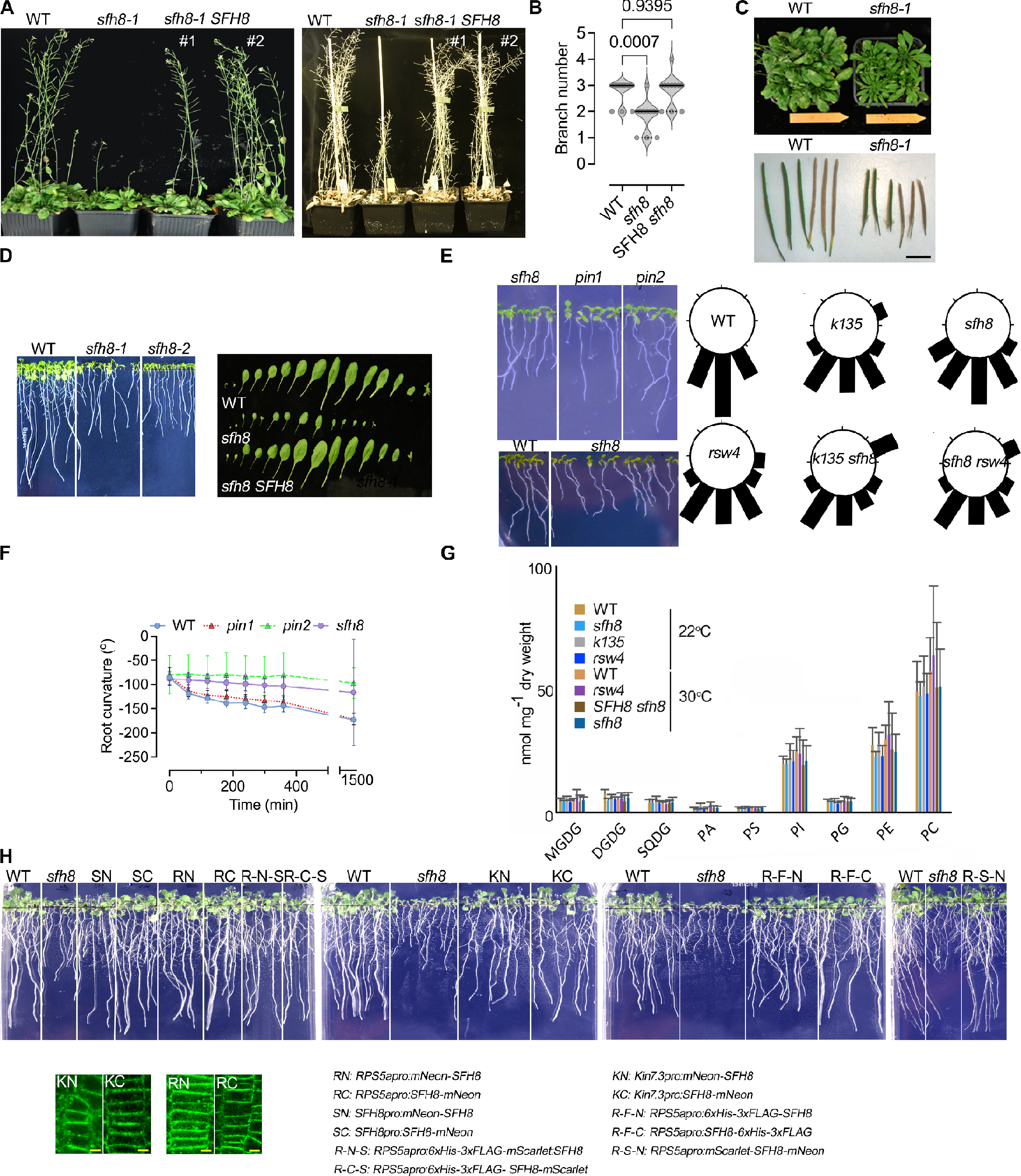

Supplement: S11 Fig — (A) Phenotype of adult plants from sfh8-1 (sfh8), and sfh8-1 SFH8 (proSFH8:SFH8-mNeon), which rescues sfh8-1 (21 and 60 DAG). (B) Branch number from adult WT, sfh8-1, or sfh8-1 SFH8 (N = 1 representative experiment, n = 9 plants). Note that this phenotype is consistent with auxin-related defects. (C) Phenotypes of adult plants from WT and sfh8-1 (upper), and their siliques (lower). Note that SFH8 mutation impacts overall growth and fecundity. Length bar, 0.8 cm. (D) Photos of WT, sfh8-1, and sfh8-2, of 5 DAG seedlings (growth in the presence of 0.5% sucrose). Right: leaf phenotype of WT, sfh8-1 (sfh8), or sfh8 SFH8. Hereafter, sfh8-1 is denoted as “sfh8,” and due to phenotypical resemblance with sfh8-2 allele is used throughout the paper. (E) Photos of WT, sfh8, and of pin1 and pin2 10 DAG seedlings. The phenotype of gravitropic defects becomes more evident by increasing growth rate with 1.5% sucrose (compare to pin1 and pin2 mutants and to WT). Right: circular plots quantitating moderate gravity perception defects of sfh8, k135, rsw4, the double rsw4 sfh8, and k135 sfh8 mutants grown vertically on plates at 11 DAG. Data are from a single representative experiment replicated 4 times (N = 4, n = 10 seedlings). (F) Quantification of a gravistimulation root tip bending assay, in which vertical plates were tilted by 90°, and the root curvature rate was determined (N = 1 representative experiment, n = 9 seedlings). The sfh8 resembled the response of KISC mutants in gravistimulation, as reported in [28]; also note the resemblance between pin2 and sfh8, in terms of slower gravistimulation response. (G) LC–MS/MS lipid species quantitative analysis in WT, sfh8, k135, sfh8 SFH8, and the rsw4 mutant (5 DAG; treated for 24 h at 28°C). Data are means ± SD (N = 5, n = 1 seedling; no significance was revealed using parametric and nonparametric tests). (H) Photos of seedlings 10 DAG expressing various fusions of SFH8 in the sfh8 background. The experiment was replicated 3 times. Th [file pbio.3002305.s022.tif]

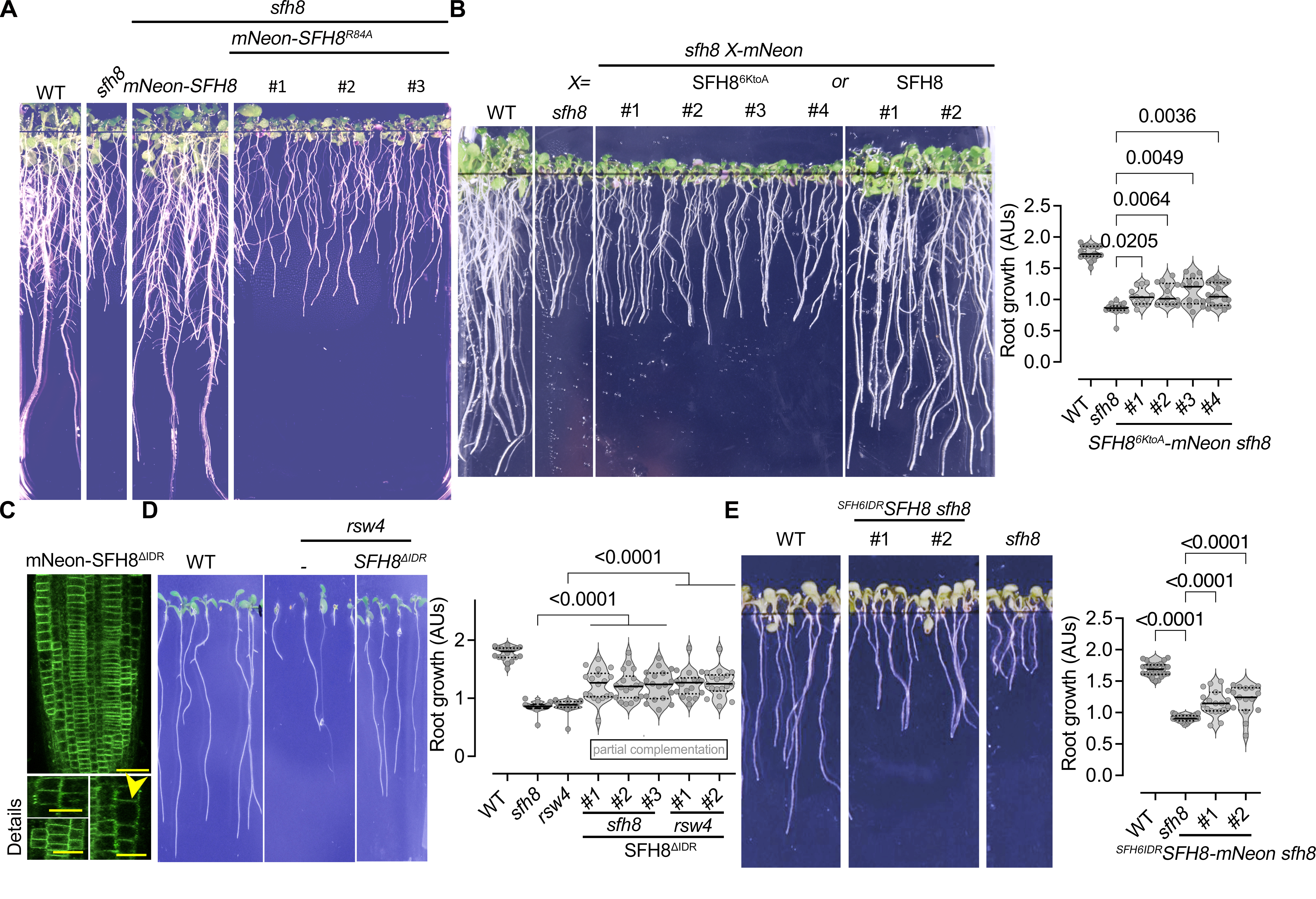

Supplement: S12 Fig — (A) Photo showing partial sfh8 rescue by RPS5a:mNeon-SFH8R84A. The experiment was replicated 3 times. (B) Photo showing partial sfh8 rescue by RPS5apro:mNeon-SFH86KtoA (10 DAG). Right: corresponding quantifications of root growth (data are means ± SD, N = 2 pooled experiments; n = 5–11 roots per experiment; p-values were calculated by ordinary 1-way ANOVA). The experiment was replicated twice. (C) Micrographs from lines expressing RPS5apro:mNeon-SFH8ΔIDR in sfh8 showing details of localization with a lack of filaments, polarization, and lack of cytoplasmic puncta formation (7 DAG). Also, note the reduced robustness of PM localization, uneven SFH8 levels, and perturbations of growth realized as reduced growth anisotropy (details; middle right cell denoted by arrowhead). The experiment replicated was 3 times. Scale bar, 40 μm and details, 20 μm. (D) Photo showing partial rsw4 rescue by RPS5apro:mNeon-SFH8ΔIDR (10 DAG). The experiment was replicated twice. Right: corresponding quantifications of root length (data are means ± SD, N = 2 pooled experiments, n = 9 seedlings per experiment; p-values were calculated by ordinary 1-way ANOVA). (E) Photo showing the partial sfh8 rescue by RPS5apro:SFH6IDRSFH8 (10 DAG). Right: corresponding quantification of root length (data are means ± SD, N = 2 pooled experiments, n = 6–12 seedlings per experiment; p-values were calculated by ordinary 1-way ANOVA). Raw data can be found in the Supporting information section (S1 Data). DAG, day after germination; PM, plasma membrane; rsw4, radially swollen 4; SFH8, SEC FOURTEEN-HOMOLOG8. (TIF) [file pbio.3002305.s023.tif]
