# Supplementary material for: SEC14-like condensate phase transitions at plasma membranes regulate root growth in Arabidopsis
Source: PLoS Biol. 2023 Sep 18;21(9):e3002305. doi: 10.1371/journal.pbio.3002305 (PMC10538751; doi:10.1371/journal.pbio.3002305)
Supplement: S1 Raw Images — (PDF) [file pbio.3002305.s010.pdf]

Figure 1

Panel A

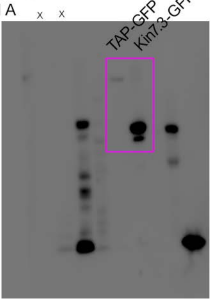

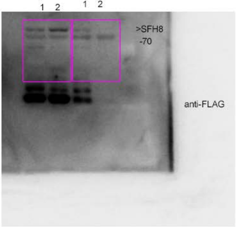

1: GFP-Kin7.3
2: GFP-TAP

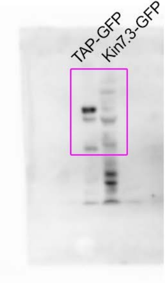

Figure 3

Panel A

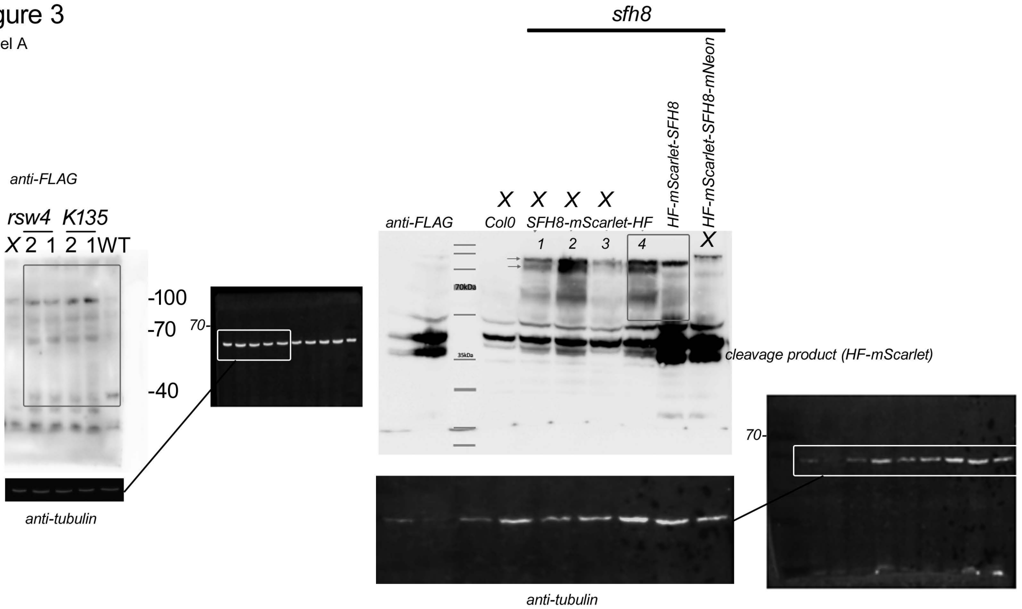

Figure S3

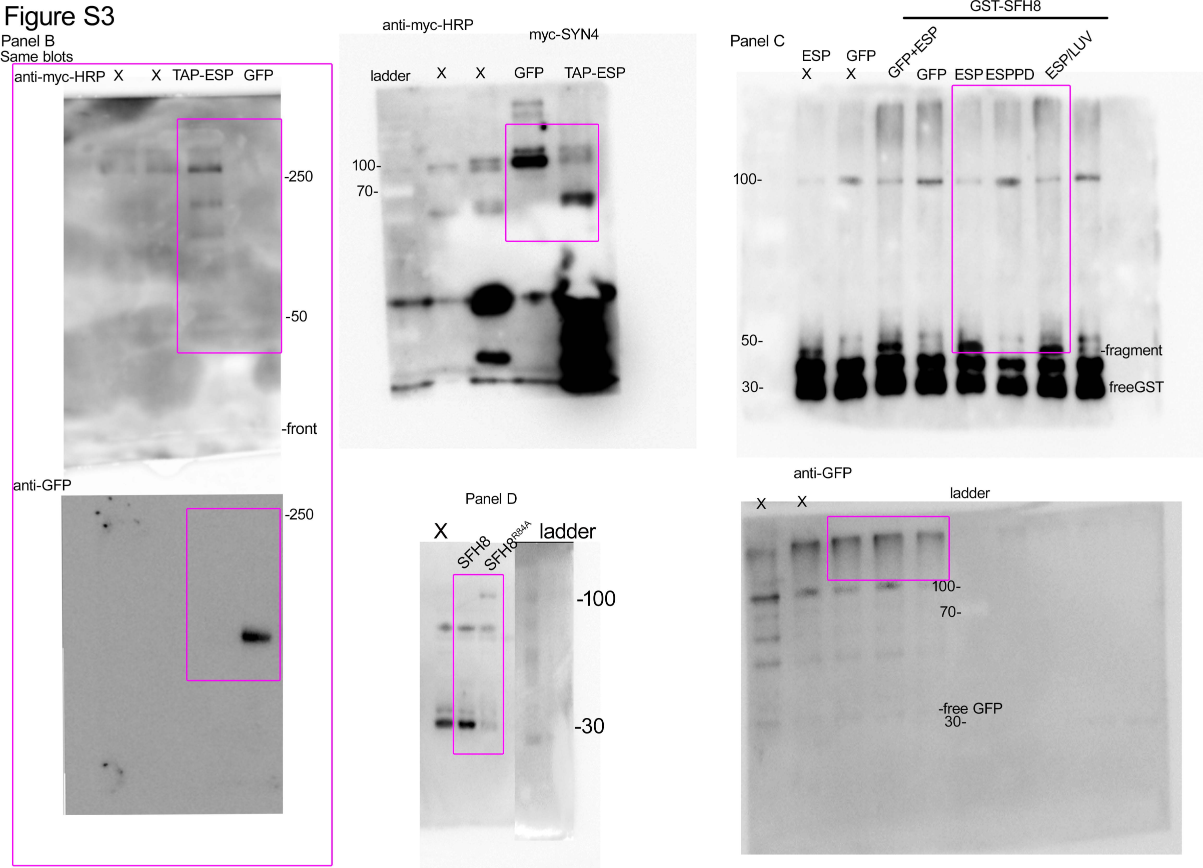
Panel B
Same blots
anti-myc-HRP X X TAP-ESP GFP
-250
-50
-front
anti-GFP
-250
anti-myc-HRP
myc-SYN4
ladder X X GFP TAP-ESP
100-
70-
GST-SFH8
Panel C
ESP X
GFP X
GFP+ESP
GFP
ESP
ESPPD
ESP/LUV
100-
50-
30-
-fragment
freeGST
anti-GFP
X X
ladder
100-
70-
-free GFP
30-
Panel D
X
SFH8
SFH8R84A
ladder
-100
-30

Figure S6

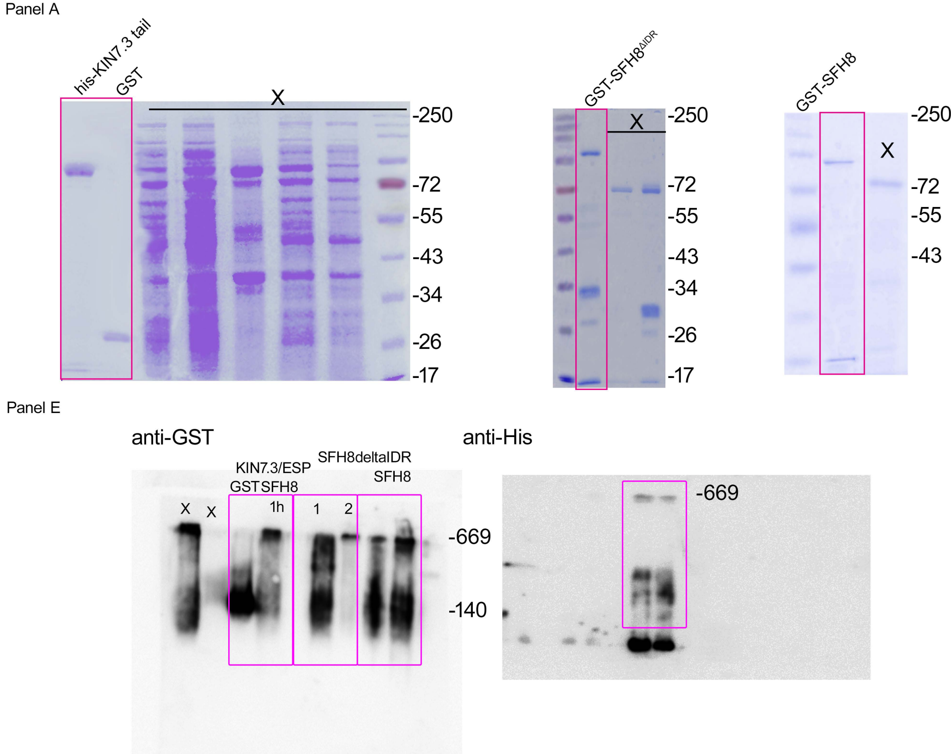
Panel A
his-KIN7.3 tail
GST
X
-250
-72
-55
-43
-34
-26
-17
GST-SFH8ΔIDR
X
-250
-72
-55
-43
-34
-26
-17
GST-SFH8
X
-250
-72
-55
-43
Panel E
anti-GST
KIN7.3/ESP
GST
SFH8
1h
SFH8deltaIDR
SFH8
X
X
1
2
-669
-140
anti-His
-669
