# Supplementary material for: SEC14-like condensate phase transitions at plasma membranes regulate root growth in Arabidopsis
Source: PLoS Biol. 2023 Sep 18;21(9):e3002305. doi: 10.1371/journal.pbio.3002305 (PMC10538751; doi:10.1371/journal.pbio.3002305)
Supplement: S1 Text — (DOCX) [file pbio.3002305.s011.docx]

***Supplemental Information***

**A Developmentally Overdue Proteolytic Switch Redefines Membrane Domains in Plants**

Liu et al., 2021

***Localization of Kin7.3 interacting proteins in stable or transient expression***

We expressed PP2C, Spc25 and POR in stable Arabidopsis Col-0 lines or N. benthamiana. Localization of these proteins is shown below (**Fig 1**). Furthermore, we identified homozygous mutants of these proteins, showing a lethal phenotype for Spc25 and POR mutants [1, 2].

The phosphatase PP2C (AP2C1), an Arabidopsis Ser/Thr phosphatase of type 2C, is a stress signal regulator that inactivates the stress-responsive MAPKs MPK4 and MPK6 [3, 4]. Mutant *ap2c1* plants produce significantly higher amounts of jasmonate upon wounding and are more resistant to phytophagous mites (*Tetranychus urticae*). Plants with increased AP2C1 levels display lower wound activation of MAPKs, reduced ethylene production, and compromised innate immunity against the necrotrophic pathogen *Botrytis cinerea*. Phosphorylation by a MAPK module regulates Kin7 superfamily members Hinkel and Tetraspore, which impinge on cell plate formation.

The Spc25 is part of the centromere assembly complex. Kin7.3 is a centromeric protein-E homolog and thus this interaction as well might be biologically relevant especially during cell division. Spc25, a component of ndc80 complex, in mouse oocytes. ndc80 complex is a conserved outer kinetochore complex, comprised of four subunits (ndc80/Hec1, nuf2, spc24 and spc25), that constitutes one of the core microtubule-binding sites within the kinetochore. One hypothesis would be that KISC during cell division is involved in microtubule stabilization on the centromeres. Among the components of the outer kinetochore complex, the four proteins in the NDC80 complex, including NDC80 [nuclear division cycle 80, also known as Hec1 (highly expressed in cancer1) in humans], NUF2 (nuclear filament‐containing protein 2), SPC24 (spindle pole body component 24) and SPC25, play critical roles in connecting spindle fibres to chromosomes. In vertebrates, the C‐terminal ends of the NUF2‐NDC80 heterodimer associate with the N‐terminal coiled‐coil domains of the SPC24‐SPC25 heterodimer [5]. Globular dimeric heads, containing the RWD (RING finger, WD repeat, DEAD‐like helicases) domain of the SPC24‐SPC25 dimer, bind to the inner kinetochore components, the KNL1‐Mis12 complex. Therefore, the NDC80 complex serves as a central ‘hub’ connecting the kinetochore complexes, which provides the ‘bridges’ between the inner kinetochore complexes and the microtubule spindles.

The PORCINO protein is a tubulin folding cofactor and its absence results in embryo lethality. The importance of Kin7.3 with PORCINO (POR) remains to be established. The TFC C ortholog, PORCINO belongs to the four *PILZ* group genes encoding orthologs of mammalian tubulin-folding cofactors (TFCs) C, D, and E, and associated small G-protein Arl2 that mediate the formation of α/β-tubulin heterodimers in vitro. PORCINO was detected in cytosolic protein complexes and did not colocalize with microtubules. Consistently, with previous reports, the *por* mutant was embryo lethal and the POR localized in the cytoplasm.


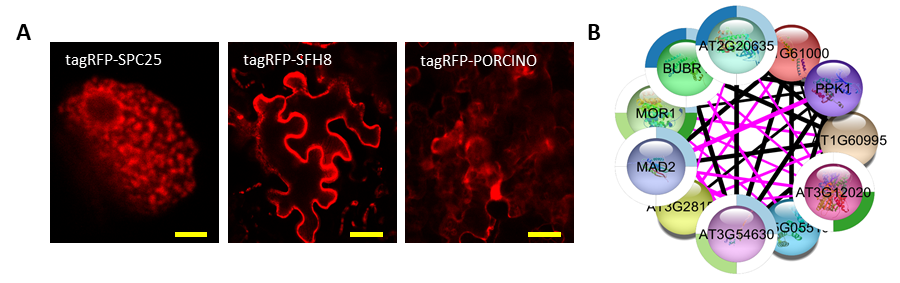


**Figure 1. Localization of Kin7.3 interactors and putative interactions of Kin7.3.**

1. Localization of SPC25, SFH8 and PORCINO tagged N-terminally with tagRPF in *N. benthamiana* transient system. Micrographs are representative of an experiment replicated three times with similar results. Scale bars, 5 um.
2. Potential Kin7.3 interactions (At3g12020).

***Cleavage of Cohesin Subunit SYN4 by Separase***

Sister-chromatid cohesion depends on cohesin, a 4-subunit protein complex that links the sisters as they are synthesized in the S phase. In anaphase when the protease separase cleaves the Scc1/ Mcd1 subunit of cohesin (or its Rec8 counterpart in meiotic cells), thereby allowing the sisters to be pulled apart by the mitotic spindle. The Arabidopsis genome encodes four kleisin subunits: the meiosis-specific SYN1 and SYN3, involved in gene expression of meiotic genes, but also expressed in somatic cells, and SYN2 and SYN4, which have been suggested to participate in mitotic cell division [6]. As we have shown previously, plants expressing tagged-SYN proteins, except the mitotic cohesin SYN4, were highly susceptible to even mild environmental changes and showed decreased fertility.

***Principle of R2D2 Probe***

R2D2 shows a diminishing green but not red fluorescence when auxin levels are high, as the conserved domain II (DII) marker (pRPS5A-driven DII fused to n3×Venus) is rapidly degraded in response to auxin. On the contrary, the pRPS5A-driven mutated DII-ntdTomato (red fluorescence) is not responding to auxin, thereby allowing the ratiometric fluorescence quantification of auxin response levels through the R2D2 [7].

**Establishment of the QCM-D Approach**

We conceived a QCM-D approach to mimic PS clusters, by designing a biorecognition surface with suspended liposomes with high PS content to test SFH8/ SFH8^ΔIDR^ binding (**Figs 7I** and **S7G**). The device surface was first covered with neutravidin (layer 1) which was used to bind specifically a 5’-biotinylated DNA (layer 2). This DNA was also modified at the 3’-end with a cholesterol moiety, further employed to anchor a liposome (layer 3). We prepared 50nm diameter liposomes with two different lipid compositions: DOPC:PI(4,5)P2 (99:1, n/n) and DOPS:PI(4,5)P2 (99:1, n/n). Finally, the SFH8 or SFH8^ΔIDR^ proteins were added (layer 4) on top of layer 3. The results presented in **Fig 7I** confirm full-length SFH8 interaction with DOPS:PI(4,5)P2 liposomes (red line, ΔF^SFH8^=~200Hz). In the absence of the IDR domain, the binding to DOPS:PI(4,5)P2 liposomes was diminished (**Fig 7I**, red line, ΔF^-IDR^=~80Hz). Besides, the binding of both proteins to DOPC:PI(4,5)P2 liposomes was relatively low (**Fig 7I**, black lines). The size of liposomes did not affect SFH8 binding, excluding the possibility that SFH8 required curvature for binding (**S7G-I Fig**).

To further address whether *in vitro* SFH8^ΔIDR^ can directly affect membrane fusion/fission, we established an *in vitro* membrane assay based on cholesterol-modified DNA zippers (hereafter “lipid-DNA-zippers” [8] (**Fig 7J**; upper left model). This minimalistic approach lacked other tethering and fusogenic proteins, thus allowing to focus on the direct SFH8 effects excluding contributions from other proteins. We adjusted concentrations of oligomers-cholesterol and used low temperature (20-22^o^C) to minimize spontaneous liposome fusion (<10%). In this assay, membrane fusion can be separated into three steps: membrane tethering in *trans*, hemifusion (lipid mixing) and fusion pore expansion. Membrane fusion results in the unification of the lipid bilayer and the intermixing of the volumes (**Fig 7J**). To test SFH8/S SFH8^ΔIDR^ -mediated membrane fusion, we used assays with low content of labelled PI(3)P, PI(4,5)P_2_ and fluorescein-containing large unilamellar vesicles (LUVs; 400 nm) with readouts based on a combination of luminal content mixing or size increments (SFH8/SFH8^ΔIDR^ showed similar affinities for the GUVs; **Fig 7J**). We selected this lipid composition to exclude differential SFH8/SFH8^ΔIDR^ binding on PS-rich membranes; recombinant mScarlet-SFH8 could bind to liposomes with this composition (**Fig 7K**). In the SFH8^ΔIDR^ samples, the average diameter of liposomes was 1 μm, in contrast to SFH8 (0.5 μm) which was below that of GST control (spontaneous fusion). Content mixing analyses showed that almost 30% in the SFH8^ΔIDR^ samples semi-fused or fused LUVs were observed (2-fold lower for SFH8l; **Fig 7L**). Again, fusion/hemifusion events in SFH8 were reduced than those of GST, suggesting that the presence of the IDR blocks fusions.

**Establishment of the DNA zippers Fusion Approach**

We adjusted concentrations of oligomers-cholesterol and used low temperature (20-22^o^C) to minimize spontaneous liposome fusion (<10%).

**References**

1. Steinborn K, Maulbetsch C, Priester B, Trautmann S, Pacher T, Geiges B, et al. The Arabidopsis PILZ group genes encode tubulin-folding cofactor orthologs required for cell division but not cell growth. Genes Dev. 2002;16(8):959-71.

2. Shin J, Jeong G, Park JY, Kim H, Lee I. MUN (MERISTEM UNSTRUCTURED), encoding a SPC24 homolog of NDC80 kinetochore complex, affects development through cell division in Arabidopsis thaliana. Plant J. 2018;93(6):977-91.

3. Bequette CJ, Hind SR, Pulliam S, Higgins R, Stratmann JW. MAP kinases associate with high molecular weight multiprotein complexes. J Exp Bot. 2018;69(3):643-54.

4. Schweighofer A, Kazanaviciute V, Scheikl E, Teige M, Doczi R, Hirt H, et al. The PP2C-type phosphatase AP2C1, which negatively regulates MPK4 and MPK6, modulates innate immunity, jasmonic acid, and ethylene levels in Arabidopsis. Plant Cell. 2007;19(7):2213-24.

5. Suzuki A, Badger BL, Haase J, Ohashi T, Erickson HP, Salmon ED, et al. How the kinetochore couples microtubule force and centromere stretch to move chromosomes. Nat Cell Biol. 2016;18(4):382-92.

6. Minina EA, Reza SH, Gutierrez-Beltran E, Elander PH, Bozhkov PV, Moschou PN. The Arabidopsis homolog of Scc4/MAU2 is essential for embryogenesis. J Cell Sci. 2017;130(6):1051-63.

7. Liao C-Y, Smet W, Brunoud G, Yoshida S, Vernoux T, Weijers D. Reporters for sensitive and quantitative measurement of auxin response. Nature methods. 2015;12(3):207-10.

8. Stengel G, Simonsson L, Campbell RA, Hook F. Determinants for membrane fusion induced by cholesterol-modified DNA zippers. J Phys Chem B. 2008;112(28):8264-74.
