# Supplementary material for: SEC14-like condensate phase transitions at plasma membranes regulate root growth in Arabidopsis
Source: PLoS Biol. 2023 Sep 18;21(9):e3002305. doi: 10.1371/journal.pbio.3002305 (PMC10538751; doi:10.1371/journal.pbio.3002305)
Supplement: Attachment — Submitted filename: PBIOLOGY-D-22-02202R1 Response to reviewers.pdf [file pbio.3002305.s025.pdf]

Response to reviewers from PBIOLOGY-D-22-02202R1

We want to thank the reviewers for their constructive criticism and useful comments on our manuscript. We have performed many additional experiments and thoroughly revised the manuscript by including new figures and making extensive and major textual amendments.

Reviewers' comments (point-by-point response):

Rev.1:

The manuscript titled "Phase transition of a SEC14-like condensate at Arabidopsis plasma membranes regulate root growth" provides a putative mechanism that how protein polarity at the plasma membrane is determined. The authors identified an SEC-like protein (SFH8) that showed polar localization in root cells which is dependent on KISC. Further analysis showed that SFH8 functioned through a phase transition that determined PIN2 polarity which is required for root growth. It seems that the authors tried to provide many approaches to support their ideas, however, the logical flow of the story should be polished before publication. The current version of the manuscript is not friendly to either audience or reviewers. For example, they provide tons of data which are actually not explained or described in the text. We have to guess what those data referred to. Thus, I would strongly suggest the authors delete those unnecessary data and strengthen the conclusion with a simplified version of the manuscript.

We have followed this advice, removed redundant data, and provided a more focused version. For example, we have removed references to the cell plate (Fig. 1D), added more data on the fusion dynamics of SFH8 clusters (Fig. 4D), and removed unnecessary information from S1 (now appended and explained below as image 1). We have also simplified (and improved) the Y2H experiments (Fig. S2; removed extensive Y2H datasets from previous Figs. S2 and S4) and reduced the information regarding the SFH8 cleavage and the corresponding controls (Fig. S3, appendix images 2, and 3). Furthermore, we have removed TEM data, including negative staining, as they did not offer much information and the quantifications were not very relevant (previous Fig. S9A). We thus now had space to include analyses for actin (Fig. S4D), and the requested mutant variants of SFH8 in different backgrounds (Fig. S12; see also below). In the FRET experiments, we removed the data regarding the dynamics of kinesin and ESP interaction at the plasma membrane, which we append below (appendix image 4). Furthermore, we removed the data regarding the specificity of SFH8 binding, which will be explored better in future work (lipid strip, previous Fig. S2B), and the clathrin binding assays (previous Fig. S10).

1. The major finding of this story is that SFH8, as a complex component with KISC, can be phase transitioned on PM which determines PIN2 localization. Thus, the authors tried to prove that phase transition of SFH8 is required for PIN2 polarity. There is some confusion in this part. We did not see a similar phenotype in the sfh8 mutant as in the pin2 mutant. pin2 mutant showed an obvious twisted root phenotype which is not displayed in either sfh8-1 or sfh8-2 (Figure S3). This majority phenotype of sfh8 is related to plant size, suggesting other mechanisms might be more important for SFH8 than regulating PIN2.

We agree that more clarifications would be required. PIN2 localization was only used as a proxy for measuring defects in polarized delivery to certain domains of the plasma membrane. We now state at line 374*"As a relevant readout here, we used PIN2 because KISC plays a role in PIN2 delivery [1], but this choice is not implying a strict link between SFH8/KISC to auxin signaling."* Furthermore, the overall defects in PIN2 in the *sfh8* mutant are rather moderate and in accordance with those described for KISC mutants in our earlier works [1]. Hence, the direct comparison to *pin2* mutant is not entirely justified, as in *sfh8* only 50% of the PIN2 is missing and is not fully absent (Fig. 6A, and quantifications on the right). The mutant of *sfh8* exhibited certain twisted root phenotypes (mainly in gravistimulation; now included with more information in Fig. S11A), easier to observe in *sfh8* mutants at slightly higher sucrose concentrations to promote growth-we now provide more evidence in this direction, and we

include an image alongside *pin2* mutants and direct comparisons. We further provide a more sensitive gravistimulation experiment, in accordance with our earlier works (using the *rsw4*; Fig. S11A). Please note that in the new version, we are more careful when discussing the links to auxin, stating explicitly that other pathways could also be affected (line 507 "*This module contributes to developmental robustness, at least partially through auxin, although we believe that this pathway has general functions in the regulation of PM domains.*". Finally, we would like to point out that other mutants with reduced PIN2 levels at the plasma membrane, for example, the *clasp* mutant ([2], lacking though polarity defects), do not show clear gravitropic phenotypes. Please see also our response for point 5.

2. The authors showed that a mutation R84A of SFH8 affects its cleavage and nano clustering, which can not rescue the sfh8 mutant phenotype. This is quite interesting. I wonder whether the cleaved or non-cleaved version of SFH8 can rescue the KISC mutant phenotype.

As per the reviewer's suggestion, we constructed new lines. The KISC mutant phenotype is partially rescued by the introduction of cleaved SFH8 (revised fig. S12C; in the *rsw4* background). We anticipate a partial rescue, as the proper timing of the transition to the uncleaved variant is important for the localization of the protein at the PM, and for the functionality (see also our response to point 4). We provide further evidence in this regard, by constructing new lines and showing that a swap of SFH8's IDR with that of SFH6 which condensates little (Fig. 5D), cannot fully rescue *sfh8* (Fig. S12D). This finding supports the idea that SFH8 condensation at the plasma membrane is functionally important.

3. Interestingly, SFH8 aligned with KISC in a filament structure. Is this aligned with the microtubule or microfilament? The authors provided some data, but it is unclear to me. This is quite important to explain how PIN2 localization is affected.

SFH8 droplets can briefly attach on microtubules (bundles) or actin but on the other hand, can persist on the plasma membrane even if microtubules/actin are absent (experiments described in Fig. S4B; "*Furthermore, amiprophos-methyl (APM) that disassembles MTs (10 nM; [1]) did not significantly alter KIN7.3 localization at the PM, although a small part of KIN7.3 filaments and in particular their edges remained attached in some cases in bundled MTs (**S4B Fig**; ~10%). This finding suggests that KIN7.3 localization at the PM does not depend on MTs. In Arabidopsis roots, SFH8 filaments were short (<0.5 μm) and insensitive to APM treatment; ESP decorated similar filaments as shown in root cells expressing GFP-tagged ESP under an inducible promoter driving expression at KIN7.3 domains (KIN7.3pro>XVEpro>GFP-ESP/RPS5apro:SFH8-mScarlet; **S4B and C Figs**). Actin depletion through pharmacological inhibition (lantraculin B), also, did not alter SFH8 localization or clustering at the PM as shown in transgenic lines co-expressing LifeAct-mCherry (Ubi10pro:LifeAct-mCherry) with mNeon-SFH8 (SFH8pro:mNeon-SFH8; **S4D Fig**). Furthermore, SFH8 did not colocalize with actin filaments at the PM (**S4D Fig**, right). These results suggest that SFH8 and KISC at the PM do not remain attached to MTs or actin.*". Careful imaging through TIRFM showed that SFH8 molecules show a transient attachment on microtubules which gives rise to only partial alignment/colocalization (now quantified, as less than 10% of the cases examined). We have evidence that SFH8 clusters can show hopping behavior on microtubules and can reside there for some time (appendix image 5). As per the reviewer's suggestion, we constructed lines co-expressing lifeact-mCherry (under Ubi10pro, using our previous assays in [3]) with mNeon-SFH8 (under its own promoter, SFH8pro) showing that mNeon-SFH8 cannot align with actin microfilaments (Fig. S4D). Furthermore, the drug latrunculin did not influence the localization of SFH8 or its clustering, thus speaking against an important role for actin in the initial localization of SFH8 and/or its clustering (Fig. S4D). Altogether, our results suggest that the KISC/SFH8 filaments are likely independent of the cytoskeleton-broadly speaking. Again, we thank the reviewer for the excellent suggestion in this direction, as we plan to study the effect of SFH8 clusters on actin/MTs in detail soon. We should also note that we have recently established that LLPS affects actin nucleation at the plasma membrane [3], and thus this field is within our interests.

4. IDR is important for LLP in many cases. However IDR-deleted version of SFH8 can partially rescue the phenotype of the sfh8 mutant. Does this mean phase transition is not important for SFH8's function as the cleavage, since SFH8-R84A can not rescue the phenotype?

The filamentous transition of SFH8 is of utmost importance for the proper delivery of polar proteins but the droplet phase is also required for the depletion zones of, for example, PIN2 (reported in Fig. 7D). If this phase does not exist, proteins like PIN2 likely mislocalize. This is a new finding in (plant) biology, and we plan to pursue it further. Our findings also provide support for suggestions of the presence of an “IDR fence” at the plasma membrane, promoted by steric pressure that reduces endocytosis and can promote protein accumulation at polar domains [4, 5]; this is consistent with SFH8, which is a polyelectrolyte [6]. We discuss these points succinctly (discussion). The uncleavable SFH8 (R84A) cannot rescue the phenotype, confirming that the phase transition is important. However, the uncleaved full-length SFH8 is also important, as in its clustered state it is required for normal development. To further reconcile these results, we swapped the SFH8 IDR for that of SFH6 which is not condensing well, providing evidence that it could promote moderate rescue to the phenotype (see also a response to point 2).

5. It is clear that PIN2 protein abundance is strongly affected in the sfh8 mutant. This can not just be ignored. All the defects can be explained simply by the low protein level of PIN2, not by mislocalization.

Likely yes, but other mutants with reduced PIN2 levels such as *clasp* do not share this phenotype (refs. [2, 7] and the retracted [8]). The reduced abundance of PIN2 derives from the reduced maintenance of PIN2 at the plasma membrane or other mechanisms. We found that *PIN2* mRNA levels are not reduced in *sfh8* or KISC mutants (Fig. S13). We have also found that indeed protein levels of PIN2-GFP or the native PIN2 (detected by anti-PIN2 in plasma membrane fractions, obtained by a protocol we used before [9]) are reduced in *sfh8* and PIN2 is less stable in *sfh8* (image 6). We assume that these effects are due to the reduced protein delivery (as we also observed an increase of intracellular signal in endosomes). Please see the same effect for PIN1, as shown in the previous version of the manuscript (Fig. S8C). Please note that the assay in image 6 is not exhaustive and would require extensive efforts to pinpoint the pathway leading to PIN2 destabilization; these efforts are much beyond our scope here but likely we will try to figure out details of the pathway soon given that proteolysis is within our interests. We can only speculate for now that if PIN2 is not properly inserted at the plasma membrane, it may have a higher turnover rate.

There are many errors in this manuscript. For example:In line 52, what is "though although"? In line 88, the S2A fig should be S3A fig.Fig S1F is not explained in the manuscript. Why did they show this data? Again, the authors have to reorganize the manuscript, delete unnecessary information, and explain the data well. I would consider it if they could provide a simple and neat version of the manuscript.

We agree and we think we have resolved the writing issues. Fig S1F showed the complementation of *k135* by the constructs tagging KIN7.3. Please see the appended images below for further details (1-4).

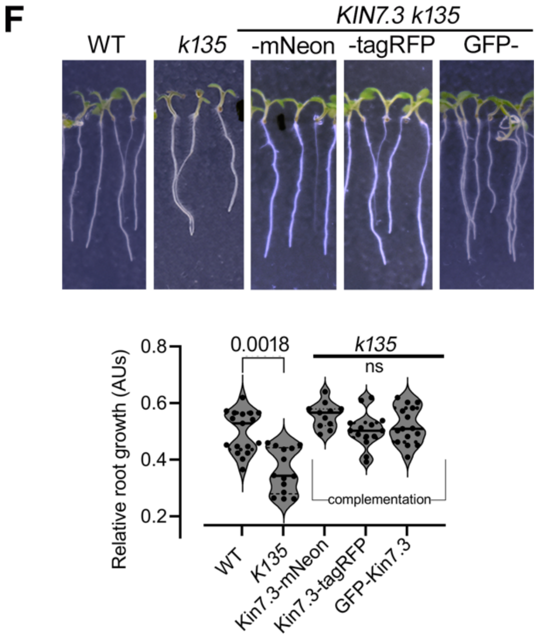

Image 1. S1F (old version). Rescue of *k135* root phenotype by various KIN7.3 fusions: *KIN7.3pro:GFP-KIN7.3*, *RPS5apro:KIN7.3-tagRFP*, or *RPS5apro:KIN7.3-mNeon*; quantification of root length (right). Data are means ± SD (N=5, n=5 roots; p-values were calculated by a 2-tailed *t*-test).

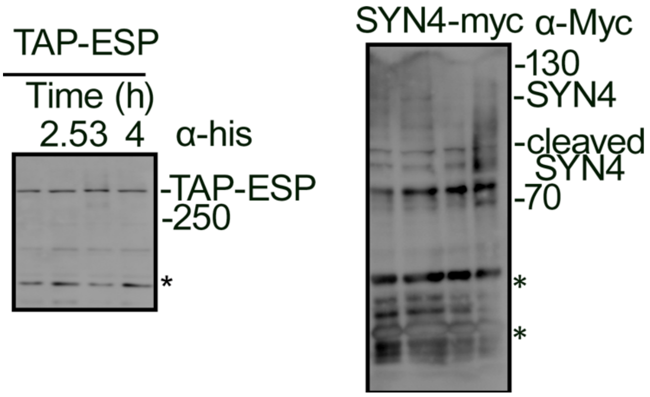

Image 2. The assay is representative of an experiment that was replicated three times. Right: increasing incubation time (2-4 h) led to the formation of additional cleavage fragments and smearing of SYN4-myc, suggesting that ESP may cleave SYN4 at multiple sites with differential preferences. Asterisks indicate non-specific bands. These blots have been removed from Fig. S3.

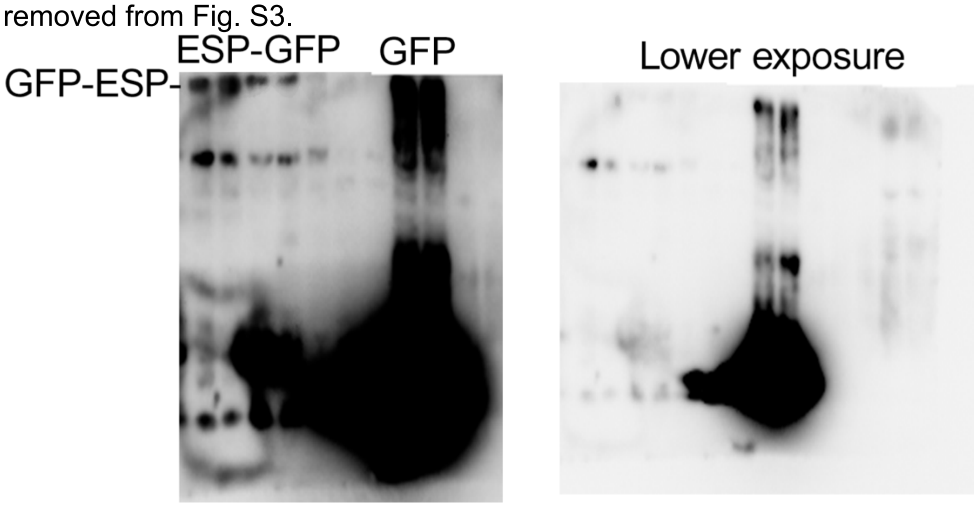

Image 3. Comparison of free GFP samples signals to that of different variants of ESP. The right shows lower exposure. Note that the GFP outcompetes the signal of GFP-ESP, and that is why we decided to show only GFP-ESP variants and their controls in Fig. S4.

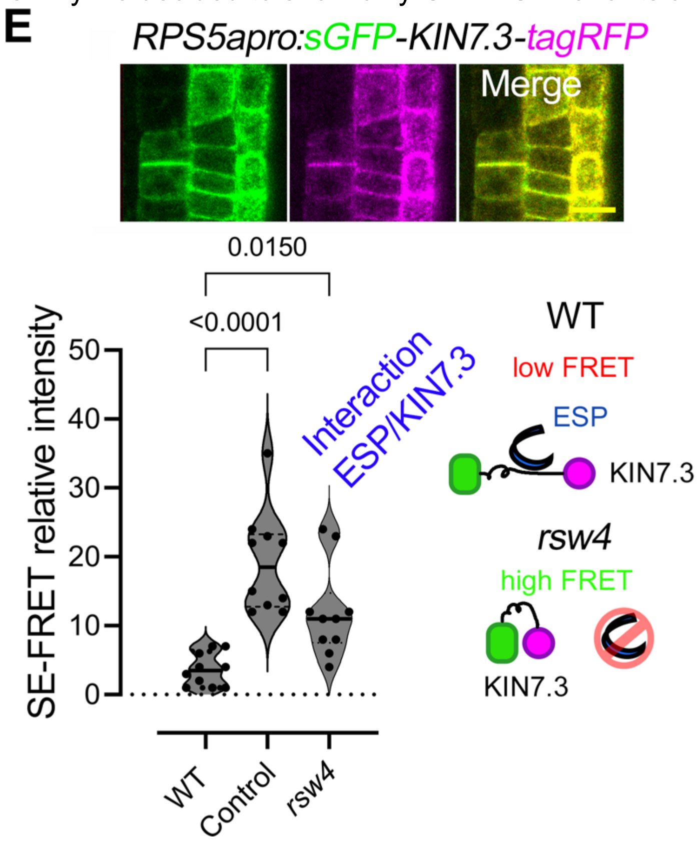

Image 4. KIN7.3 interacts with ESP at the PM. SE-FRET experiments at the PM show that N and C termini of KIN7.3 fold over in the absence of ESP (*rsw4*; see also [1] for a similar situation upon MT binding), suggesting also that ESP/KIN7.3 interact at the PM. Scale bar (micrograph), 5 µm. “control”: *35Spro:sGFP-tagRFP* expressing lines (yielding high FRET). Data are means±SD (N=3, n=3 roots; Wilcoxon).

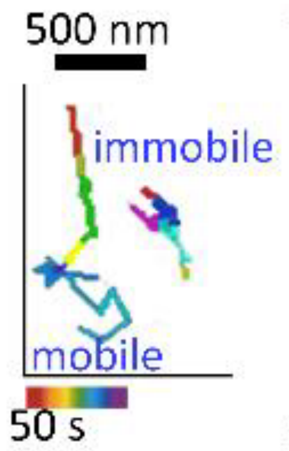

Image 5. Tracking analysis of an SFH8-cluster showing a part with directional diffusion. When diffusing SFH8 clusters were nearby pre-established KISC filaments, they showed a “hop” diffusion consistent with anchoring or sterical entrapment on MTs. Note the immobile part that shows directional diffusion. Time and spatial scale are shown (500 nm, 50 s corresponding to 500 frames).

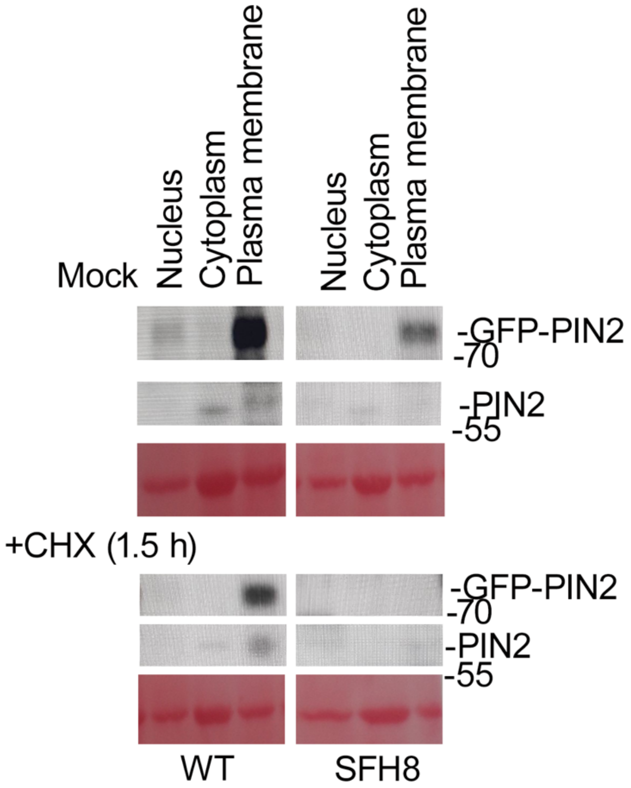

Image 6. PIN2 protein levels in fractionated roots (nucleus, cytoplasm and plasma membrane fractions), in the presence or absence of cycloheximide.

Rev. 2: Farhah Assaad – note that this reviewer has signed his review. Please use uploaded word file if possible, non-formatted text is below.

In this manuscript, Liu et al. address the role of liquid to liquid phase separation (LLPS) and liquid to solid phase transitions at the plasma membrane (PM) in Arabidopsis. This study builds on the characterization of KISC, a multiprotein complex consisting of Separase and 3 homologs of Kinesin 7 (including KIN 7.3; Moschou et al., Dev Cell 2016). The study by Liu et al. starts with interaction screens or assays (yeast two-hybrid, co-immunoprecipitation and ratiometric bimolecular fluorescence) with KIN7.3 as bait. This identified SFH8, a previously uncharacterized SEC 14 like lipid transfer protein, as a KIN7.3 interaction partner.The authors report that* SFH8 tethers KISC on the plasma membrane.* In turn, KISC promotes SFH8 polarization.
* SFH8 has an Intrinsically Disordered Region (IDR) at its N-terminus, spanning 1-84aa.* KISC cleaves SFH8 at the aa residue R84 (potential cleavage site). This releases two fragments of SFH8: (i) an N-terminal SFH8IDR and (ii) a C-terminal SFH8ΔIDR.* Full length SFH8, an uncleavable SFH8 variant and the cleaved N-terminal SFH8IDR can be seen to form cytoplasmic puncta.* In contrast, the cleaved C-terminal SFH8ΔIDR colocalizes with KISC and PIN2 at the plasma membrane and has a striated or filamentous appearance on confocal, TIRF and electron micrographs.* The cleaved C-terminal SFH8ΔIDR allows the association and polar delivery of PIN2 on the PM.* In contrast, full length SFH8 or the N-terminal SFH8IDR block PIN2 delivery.* SFH8 and KISC mutant alleles, truncations or variants have defects in (i) root growth, (ii) gravitropism, (iii) auxin distribution and (iv) PIN2 polarity.

This is a highly novel, impressive and interesting study. There are a massive number of different experimental approaches, including targeted mutagenesis, the catGranule algorithm,

FRAP rates, quantitative proximity ligation (PLA) assays and an in vitro membrane fusion assay based on cholesterol-modified DNA zippers. The manuscript makes inroads into unchartered territory.

However, the manuscript was somewhat overwhelming to read. More importantly, it is not uniformly clear that the main conclusions are supported by the dataset.

Major concerns:

1. The authors interpret SFH8IDR puncta as condensates or clusters formed via LLPS. Conversely, C-terminal SFH8ΔIDR filaments are interpreted as being formed by a liquid to solid phase transition of the SFH8 condensate, initially in the LLPS form. While this view is likely correct, it would be important to start by exploring and excluding alternative hypotheses. KISC, for example, is known to control microtubule dynamics (Moschou et al., Dev Cell 2016). The authors exclude microtubule association as underlying the striated, filamentous appearance of SFH8 on the counts of (i) colocalization and (ii) the impact of microtubule depolymerizing drugs (S4A, B Figs). This argument could be tightened and the role of actomyosin filaments excluded as well. Once alternative hypotheses are excluded, the authors could further strengthen their case by outlining the properties of LLPS and grouping the data in the manuscript that exhibit these properties. Key features of liquid-like compartments include: (a) spherical appearance, (b) fusing with each other upon touching, (c) drip in shear flows and (d) fluid internal components (Cuevas-Velazquez & Dinneny, 2018, Curr Opin Plant Biol; additional criteria such as defining saturation concentrations are described in Alberti et al., 2019, Cell).

As per the reviewer's suggestion, we constructed lines co-expressing lifeact-mCherry (under Ubi10pro) with mNeon-SFH8 (under its own promoter, SFH8pro; Fig. S4D). We could not see the co-alignment of SFH8 filaments with actin (please see also the response to reviewer 1). Furthermore, the drug latrunculin did not influence the localization of SFH8 or its clustering, thus speaking against an important role for actin in the initial localization of SFH8 and/or its clustering (Fig. S4D). Please see revised lines 234-237. Altogether, our results suggest that the KISC/SFH8 filaments are likely independent of the cytoskeleton. We cannot exclude, however, that the finer dynamics of these filaments are affected by SFH8 clusters; please see also image 5, showing a transient directional movement of an SFH8 cluster along MT (decorated also by KIN7.3). These dynamics though are beyond our scope here and would require extensive efforts to define protocols for measuring the fine dynamics of MTs/actin at the plasma membrane in meristematic cells where these filaments are not bundled enough.

We think that the designated approaches for analyzing cytoplasmic condensates may not be the most adequate here. Processes such as dripping and those described in the publications above likely are not so relevant for membrane-bound condensates, as they deform through the physical interfacing with the underlying lipids (known as wetting; for relevant discussions, please see refs. [6, 10-14]). We though provide some further examination of condensates' deformities (Fig. 4D, grouping into categories and measuring circularity, i.e., spherical appearance). Regarding saturation concentrations (Csat), please note that these in vivo can be skewed because of the nucleating lipids functioning by reducing the Csat by an order of magnitude, if not more. The underlying mechanism involves dimensionality reduction and reduced diffusion (reported for condensates in animals, like those of Whi3 [15]). We should note here that by no means now we can exclude the possibility of emulsion formation or other complex biophysical processes, that we are currently investigating. Shear flow on surfaces is affected by the surface itself and cannot be modelled through dripping efficiently [16], even though the surfaces we study are more like a gel rather than a solid. Along these lines is also the recent pre-print, showing that condensates interfacing with membranes can affect the ordered lipids [17].

2. In Figure 1D the authors fail to distinguish between cross walls versus cell plates. Furthermore, they use a marker (FM4-64) that does not discriminate between the middle of

the cell plate versus its leading edge. Cross walls or emerging plasma membranes formed during cytokinesis are not crucial to the story line, so one could simply delete these panels.

We agree, and for the sake of brevity, we removed the data.

3. The introduction and discussion are very short and the relevant literature not adequately reviewed. LLPS has been implicated in viral assembly and human disease. In plants, LLPS has been implicated in metabolism, repair, sorting and in responses to biotic and abiotic stress (b; doi:10.1038/s41586-019-0880-5; doi:10.1016/j.cell.2020.02.045; doi:10.1038/s41586-021-03572-6; doi:10.1016/j.cell.2020.09.010). The importance of the IDR in the plant literature could also be better outlined. To review the literature would highlight the novelty of the findings described in this manuscript.

We agree and have extended both including the suggested literature.

4. The manuscript is very difficult to follow in its current shape. It would benefit from streamlining. In addition, the flow of logic should be tightened. As a suggestion, the structure could be outlined as follows: (i) identification and characterization of SFH8 (ii) cleavage of SFH8 (iii) filamentous versus punctate appearance of SFH8 full length, variants or fragments (iv) the case for LLPS or liquid to solid transitions (v) the role of LLPS in PIN polarity (including Fig. 2F, 2G), auxin distribution (Fig. 9) and plant development, including gravitropism (Figs 2G, S3A). The discussion should start with a recapitulation of the results. A graphical abstract would also help the reader.

We agree and the manuscript has already been rewritten following this order.

Minor comments:

5. The figures are difficult to navigate. It would help if they could be structured in a grid-like manner and if the reader could move through them either from left to right or from top to bottom. Different annotations (as opposed to the same annotation in a different color) such as arrows, arrowheads or asterisks should be used to label structures and salient features consistently and clearly throughout the manuscript. The text font on the figures is too large, such that the labels are at times longer than the panels. Furthermore, the labels have too much information; promoters, for example, could be in the legend and the minimal relevant designator on the panel.

We now changed the figures according to the structure suggested by the reviewer and further polished the callout and label to be more uniform and neater.

6. In Figure 2A the authors refer to the role of SFH8 in the "modulation of development" (an action subtitle; would a descriptive subtitle not be preferable?); here they should stay closer to their observations and describe more specifically the impact on root growth.

Please see the new title, as according to the structure of the manuscript, it was changed to "*SFH8 recruits KISC at the PM and KISC regulates SFH8 polarity*".

7. 7 In Fig 2C the authors write "The depletion of sfh8 leads to a reduced meristem size"; it is not clear what the arrow heads point to, nor how meristem size was assessed.

Now: *"(C) Confocal micrographs of root meristems (5 DAG; red signal: stained cell walls with propidium iodide), and quantifications of WT, sfh8, or KISC mutants' meristem sizes (n=19; ordinary ANOVA). The arrowheads indicate the meristem (from QC to the first elongating cell). Micrographs are representative of an experiment replicated ten times. Scale bars, 50 µm."*

8. In Fig 2D the authors write "The reduction of sfh8 meristem size is due to compromised cell divisions". However, the authors have not monitored cell expansion as a function of distance from the QC/ i.e. exit from the meristem, which also influences meristem size. The conclusion could be turned around to "compromised cell division contributes to the reduction in meristem size".

Now: "(*D*) *Confocal micrograph of the "Cycle Tracking in Plants" marker (CyTRAP, tracking S and M phases of the cell cycle in root cells) in WT and sfh8 (7 DAG). The compromised cell divisions likely contribute to the reduction in meristem size. Scale bars, 50 µm. Lower: quantification of S and M phase (N=3 pooled experiments, n=10 roots; p-values were calculated by a paired t-test)."*

9. Fig 2G is all important as it shows an impact of KISC disruption via transient over expression of the KIN7.3 tail on gravitropism; this should be quantified as shown for other mutants in S3A Fig (circular plots showing moderate gravity perception defects of sfh8, k135, rsw4, the double rsw4 sfh8, and k135 sfh8 mutants).

Now: thanks for the suggestions; quantifications have been added (Fig. 2D and .csv file including the control line).

10. It would simplify the main figures considerably if the authors could focus on region 3 and relegate other regions of the root to the supporting information files.

We agree, but there are instances that this cannot be done and can even lead to confusion. For example, in Fig 1 it is important to show the dynamics of localization, in Fig 3 the dynamics of cleavage, and in Fig 7 region 1 is of utmost importance.

11. The legends of the figures 3B, 4A, S1B, S1C, S2A, S3A, S6A, S9B, S10A state that experiments were replicated multiple times. However, the exact number of replicates is not indicated.

Corrected throughout the text and in all instances.

REFERENCES

1. Moschou PN, Gutierrez-Beltran E, Bozhkov PV, Smertenko A. Separase Promotes Microtubule Polymerization by Activating CENP-E-Related Kinesin Kin7. Dev Cell. 2016;37(4):350-61.

2. Ambrose C, Ruan Y, Gardiner J, Tamblyn LM, Catching A, Kirik V, et al. CLASP interacts with sorting nexin 1 to link microtubules and auxin transport via PIN2 recycling in Arabidopsis thaliana. Dev Cell. 2013;24(6):649-59.

3. Liu C, Mentzelopoulou A, Muhammad A, Volkov A, Weijers D, Gutierrez-Beltran E, et al. An actin remodeling role for Arabidopsis processing bodies revealed by their proximity interactome. Embo J. 2023:e111885.

4. Ritchie K, Iino R, Fujiwara T, Murase K, Kusumi A. The fence and picket structure of the plasma membrane of live cells as revealed by single molecule techniques (Review). Mol Membr Biol. 2003;20(1):13-8.

5. Gollapudi S, Jamal S, Kamatar A, Yuan F, Wang L, Lafer EM, et al. Steric pressure between glycosylated transmembrane proteins inhibits internalization by endocytosis. Proc Natl Acad Sci U S A. 2023;120(15):e2215815120.

6. Mondal S, Cui Q. Coacervation of poly-electrolytes in the presence of lipid bilayers: mutual alteration of structure and morphology. Chem Sci. 2022;13(26):7933-46.

7. Ruan Y, Halat LS, Khan D, Jancowski S, Ambrose C, Belmonte MF, et al. The Microtubule-Associated Protein CLASP Sustains Cell Proliferation through a Brassinosteroid Signaling Negative Feedback Loop. Current Biology. 2018;28(17):2718-29.e5.

8. Dhonukshe P, Weits DA, Cruz-Ramirez A, Deinum EE, Tindemans SH, Kakar K, et al. A PLETHORA-auxin transcription module controls cell division plane rotation through MAP65 and CLASP. Cell. 2012;149(2):383-96.

9. Michalopoulou VA, Mermigka G, Kotsaridis K, Mentzelopoulou A, Celie PHN, Moschou PN, et al. The host exocyst complex is targeted by a conserved bacterial type-III effector that promotes virulence. Plant Cell. 2022;34(9):3400-24.

10. Agudo-Canalejo J, Schultz SW, Chino H, Migliano SM, Saito C, Koyama-Honda I, et al. Wetting regulates autophagy of phase-separated compartments and the cytosol. Nature. 2021;591(7848):142-6.

11. Botterbusch S, Baumgart T. Interactions between Phase-Separated Liquids and Membrane Surfaces. Appl Sci (Basel). 2021;11(3).

12. Kusumaatmaja H, May AI, Feeney M, McKenna JF, Mizushima N, Frigerio L, et al. Wetting of phase-separated droplets on plant vacuole membranes leads to a competition between tonoplast budding and nanotube formation. Proc Natl Acad Sci U S A. 2021;118(36):e2024109118.

13. Kusumaatmaja H, May AI, Knorr RL. Intracellular wetting mediates contacts between liquid compartments and membrane-bound organelles. J Cell Biol. 2021;220(10).

14. Morin JA, Wittmann S, Choubey S, Klosin A, Golfier S, Hyman AA, et al. Sequence-dependent surface condensation of a pioneer transcription factor on DNA. Nature Physics. 2022;18(3):271-6.

15. Snead WT, Jalihal AP, Gerbich TM, Seim I, Hu Z, Gladfelter AS. Membrane surfaces regulate assembly of ribonucleoprotein condensates. Nat Cell Biol. 2022.

16. Kurotani Y, Tanaka H. A novel physical mechanism of liquid flow slippage on a solid surface. Sci Adv. 2020;6(13):eaaz0504.

17. Wang H-Y, Chan SH, Dey S, Castello-Serrano I, Ditlev JA, Rosen MK, et al. Coupling of protein condensates to ordered lipid domains determines functional membrane organization. bioRxiv. 2022:2022.08.02.502487.
