# Supplementary material for: SEC14-like condensate phase transitions at plasma membranes regulate root growth in Arabidopsis
Source: PLoS Biol. 2023 Sep 18;21(9):e3002305. doi: 10.1371/journal.pbio.3002305 (PMC10538751; doi:10.1371/journal.pbio.3002305)
Supplement: Attachment — Submitted filename: PBIOLOGY-D-22-02202R2_Response to reviewers.pdf [file pbio.3002305.s026.pdf]

PBIOLOGY-D-22-02202R2
Response to reviewers:
We want to thank both reviewers for their time, criticism, and useful comments. We have now addressed all their comments.
<u>Reviewers' comments (point-by-point response):</u>
Rev. 1:
The revised version of the manuscript is indeed much improved. The logic flow is much clear. A few minor concerns need to be corrected.
1. Figure 1B, for the Free YFP/RFP part, the right figure is not the merged image. Please confirm.
We have now fixed this micrograph and added the merged one.
2. Figure 2A (right panel), is a bit confusing how the N-truncated and C-cleavage are detected in the same gel. Please clarify.
We now have added an arrow and provided an explanation in figure legends.
3. Figure 10 is unnecessary. It did not help the whole story to show that auxin is involved without any possible mechanism. The current story is already complicated.
As both the reviewers find the story complicated, we agree, and we have removed Fig. 10.
4. An appropriate model might help to understand the story better.
We now include a graphical abstract (new Fig. 10).
Rev. 2:
This is a review of a revised manuscript. The authors have made some effort to address reviewer comments, but have not fully embraced our criticism, which was meant to be constructive. The manuscript addresses the role of liquid to liquid phase separation (LLPS) and liquid to solid phase transitions at the plasma membrane (PM) in Arabidopsis. This study builds on the characterization of KISC, a multiprotein complex consisting of Separase and 3 homologs of Kinesin 7 (including KIN 7.3; Moschou et al., Dev Cell 2016). The study by Liu et al. starts with interaction screens or assays (yeast two-hybrid, co-immunoprecipitation and ratiometric bimolecular fluorescence) with KIN7.3 as bait. This identified SFH8, a previously uncharacterized SEC 14 like lipid transfer protein, as a KIN7.3 interaction partner. The concerns listed in the review are in abbreviated form in black and my comments on the rebuttal is in blue (now indicated as 'response').
<u>Note added by the authors</u>: reviewer's responses are indicated with green.
Major concerns:
1. The authors interpret SFH8 IDR puncta as condensates or clusters formed via LLPS. Conversely, C-terminal SFH8 IDR filaments are interpreted as being formed by a liquid to solid phase transition of the SFH8 condensate, initially in the LLPS form. While this view is likely correct, it would be important to start by exploring and excluding alternative hypotheses.
Response: The authors have done a thorough job on this first comment and it would be important to make the considerations in the rebuttal apparent in the text.
We agree and we now include the most relevant parts of the rebuttal in the main text: *As a cautionary note here, we did not examine other parameters used for cytoplasmic condensates, such as dripping or saturation concentrations as membrane-bound condensates, deform through the physical interfacing with the underlying lipids (the process known as wetting; [1-4]).*
2. Response: Reference to cell plates or cross walls has been deleted, as requested.
Ok.
3. The introduction and discussion are very short and the relevant literature not adequately reviewed.
Response: The new literature includes a reference to Dhonukshe P, et al., A PLETHORA-auxin transcription module controls cell division plane rotation through MAP65 and CLASP. Cell. 2012 149(2):38396. doi: 10.1016/j.cell.2012.02.051, which has been retracted: Cell. 2013 Nov 21;155(5):1189. PMID: 22500804. It would be good to check the literature more carefully.
We thank the reviewer for advising on this end. We had designated in our rebuttal this reference as retracted: *Likely yes, but other mutants with reduced PIN2 levels such as clasp*

do not share this phenotype (refs. [5, 6] and the retracted [7]). Furthermore, this reference is not included in the text of our manuscript.
4. The manuscript is very difficult to follow in its current shape.
Response: The manuscript is still daunting to read and follow: it requires an inordinate amount of time and attention from the reader, which is simply not forthcoming. There is still no graphical abstract.
We have now removed a significant amount of data through the exclusion of Fig. 10 (auxin-related signalling; and associated Fig. S13) and included a graphical abstract (new Fig. 10, along with a legend). We are happy to follow the Editors' advice on whether this graphical abstract can be converted to a striking image and be removed from the main text. Furthermore, we have reduced by 10% the character count in all sessions of the manuscript, by using more concise writing (abstract, 12 words reduction; main text, 6,283 words vs. 7,000 in the previous version). We have further removed redundant information reducing the text of supplemental information, and legends (by 20%).
Minor comments:
1. The figures are difficult to navigate. It would help if they could be structured in a grid-like manner and if the reader could move through them either from left to right or from top to bottom. Different annotations (as opposed to the same annotation in a different color) such as arrows, arrowheads or asterisks should be used to label structures and salient features consistently and clearly throughout the manuscript. The text font on the figures is too large, such that the labels are at times longer than the panels. Furthermore, the labels have too much information; promoters, for example, could be in the legend and the minimal relevant designator on the panel.
Response: The figures are still not grid-like in their organization; they are still too busy and have single designations for multiple panels. The text on the panels is still conveys too much information.
We have now provided grid-like panels (left-to-right orientation) and provided more designators for the panels. We did our best to reduce the information in the panels (e.g., by removing construct names and redundant information).
2. In Figure 2A the authors refer to the role of SFH8 in the "modulation of development" (an action subtitle; would a descriptive subtitle not be preferable?); here they should stay closer to their observations and describe more specifically the impact on root growth.
Response: OK.
3. In Fig 2C the authors write "The depletion of sfh8 leads to a reduced meristem size"; it is not clear what the arrow heads point to, nor how meristem size was assessed.
Response: Here the manner in which the first elongating cell was defined is unclear.
We now include the statement: "*showing >50% increase of size along the proximodistal axis*" (see also Fig. 9E). Furthermore, we corrected the offset of the corresponding yellow arrowheads during image preparation.
4. In Fig 2D the authors write "The reduction of sfh8 meristem size is due to compromised cell divisions". However, the authors have not monitored cell expansion as a function of distance from the QC/ i.e. exit from the meristem, which also influences meristem size. The conclusion could be turned around to "compromised cell division contributes to the reduction in meristem size".
Response: OK.
5. Fig 2G is all important as it shows an impact of KISC disruption via transient over expression of the KIN7.3 tail on gravitropism; this should be quantified as shown for other mutants in S3A Fig (circular plots showing moderate gravity perception defects of sfh8, k135, rsw4, the double rsw4 sfh8, and k135 sfh8 mutants).
Response: OK.
6. It would simplify the main figures considerably if the authors could focus on region 3 and relegate other regions of the root to the supporting information files.
Response: Maybe this cannot be done everywhere, but the unnecessary regions should be deleted where possible.

We agree; Fig. 7A, regions 2 and 4 and in Fig. 8F, regions 1 and 4 from SE-FRET micrographs, were removed.

7. The legends of the figures 3B, 4A, S1B, S1C, S2A, S3A, S6A, S9B, S10A state that experiments were replicated multiple times. However, the exact number of replicates is not indicated.

Response: OK.

REFERENCES

1. Agudo-Canalejo J, Schultz SW, Chino H, Migliano SM, Saito C, Koyama-Honda I, et al. Wetting regulates autophagy of phase-separated compartments and the cytosol. Nature. 2021;591(7848):142-6.

2. Botterbusch S, Baumgart T. Interactions between Phase-Separated Liquids and Membrane Surfaces. Appl Sci (Basel). 2021;11(3).

3. Kusumaatmaja H, May AI, Feeney M, McKenna JF, Mizushima N, Frigerio L, et al. Wetting of phase-separated droplets on plant vacuole membranes leads to a competition between tonoplast budding and nanotube formation. Proc Natl Acad Sci U S A. 2021;118(36):e2024109118.

4. Mondal S, Cui Q. Coacervation of poly-electrolytes in the presence of lipid bilayers: mutual alteration of structure and morphology. Chem Sci. 2022;13(26):7933-46.

5. Ruan Y, Halat LS, Khan D, Jancowski S, Ambrose C, Belmonte MF, et al. The Microtubule-Associated Protein CLASP Sustains Cell Proliferation through a Brassinosteroid Signaling Negative Feedback Loop. Current Biology. 2018;28(17):2718-29.e5.

6. Ambrose C, Ruan Y, Gardiner J, Tamblyn LM, Catching A, Kirik V, et al. CLASP interacts with sorting nexin 1 to link microtubules and auxin transport via PIN2 recycling in Arabidopsis thaliana. Dev Cell. 2013;24(6):649-59.

7. Dhonukshe P, Weits DA, Cruz-Ramirez A, Deinum EE, Tindemans SH, Kakar K, et al. A PLETHORA-auxin transcription module controls cell division plane rotation through MAP65 and CLASP. Cell. 2012;149(2):383-96.
