# Supplementary material for: SEC14-like condensate phase transitions at plasma membranes regulate root growth in Arabidopsis
Source: PLoS Biol. 2023 Sep 18;21(9):e3002305. doi: 10.1371/journal.pbio.3002305 (PMC10538751; doi:10.1371/journal.pbio.3002305)
Supplement: Attachment — Submitted filename: PLOS Biol review final.docx [file pbio.3002305.s024.docx]

**Reviewer Comments for Manuscript Number PBIOLOGY-D-22-02202R1**

In this manuscript, Liu et al. address the role of liquid to liquid phase separation (LLPS) and liquid to solid phase transitions at the plasma membrane (PM) in Arabidopsis. This study builds on the characterization of KISC, a multiprotein complex consisting of Separase and 3 homologs of Kinesin 7 (including KIN 7.3; Moschou et al., Dev Cell 2016). The study by Liu et al. starts with interaction screens or assays (yeast two-hybrid, co-immunoprecipitation and ratiometric bimolecular fluorescence) with KIN7.3 as bait. This identified SFH8, a previously uncharacterized SEC 14 like lipid transfer protein, as a KIN7.3 interaction partner.

The authors report that

- SFH8 tethers KISC on the plasma membrane.
- In turn, KISC promotes SFH8 polarization.
- SFH8 has an Intrinsically Disordered Region (IDR) at its N-terminus, spanning 1-84aa.
- KISC cleaves SFH8 at the aa residue R84 (potential cleavage site). This releases two fragments of SFH8: (i) an N-terminal SFH8^IDR^ and (ii) a C-terminal SFH8^ΔIDR^.
- Full length SFH8, an uncleavable SFH8 variant and the cleaved N-terminal SFH8^IDR^ can be seen to form cytoplasmic puncta.
- In contrast, the cleaved C-terminal SFH8^ΔIDR^ colocalizes with KISC and PIN2 at the plasma membrane and has a striated or filamentous appearance on confocal, TIRF and electron micrographs.
- The cleaved C-terminal SFH8^ΔIDR^ allows the association and polar delivery of PIN2 on the PM.
- In contrast, full length SFH8 or the N-terminal SFH8^IDR^ block PIN2 delivery.
- SFH8 and KISC mutant alleles, truncations or variants have defects in (i) root growth, (ii) gravitropism, (iii) auxin distribution and (iv) PIN2 polarity.

This is a highly novel, impressive and interesting study. There are a massive number of different experimental approaches, including targeted mutagenesis, the catGranule algorithm, FRAP rates, quantitative proximity ligation (PLA) assays and an in vitro membrane fusion assay based on cholesterol-modified DNA zippers. The manuscript makes inroads into unchartered territory.

However, the manuscript was somewhat overwhelming to read. More importantly, it is not uniformly clear that the main conclusions are supported by the dataset.

Major concerns:

1. The authors interpret SFH8^IDR^ puncta as condensates or clusters formed via LLPS. Conversely, C-terminal SFH8^ΔIDR^ filaments are interpreted as being formed by a liquid to solid phase transition of the SFH8 condensate, initially in the LLPS form. While this view is likely correct, it would be important to start by **exploring and excluding alternative hypotheses**. KISC, for example, is known to control microtubule dynamics (Moschou et al., Dev Cell 2016). The authors exclude microtubule association as underlying the striated, filamentous appearance of SFH8 on the counts of (i) colocalization and (ii) the impact of microtubule depolymerizing drugs (S4A, B Figs). This argument could be tightened and the role of actomyosin filaments excluded as well. Once alternative hypotheses are excluded, the authors could further strengthen their case by outlining the properties of LLPS and grouping the data in the manuscript that exhibit these properties. Key features of liquid-like compartments include: (a) spherical appearance, (b) fusing with each other upon touching, (c) drip in shear flows and (d) fluid internal components (Cuevas-Velazquez & Dinneny, 2018, *Curr Opin Plant Biol*; additional criteria such as defining saturation concentrations are described in Alberti et al., 2019, *Cell*).
2. In Figure 1D the authors fail to distinguish between cross walls versus cell plates. Furthermore, they use a marker (FM4-64) that does not discriminate between the middle of the cell plate versus its leading edge. Cross walls or emerging plasma membranes formed during cytokinesis are not crucial to the story line, so one could simply delete these panels.
3. The introduction and discussion are very short and the relevant literature not adequately reviewed. LLPS has been implicated in viral assembly and human disease. In plants, LLPS has been implicated in metabolism, repair, sorting and in responses to biotic and abiotic stress (doi:10.1016/j.molp.2022.05.007; doi:10.1038/s41586-019-0880-5; doi:10.1016/j.cell.2020.02.045; doi:10.1038/s41586-021-03572-6; doi:10.1016/j.cell.2020.09.010). The importance of the IDR in the plant literature could also be better outlined. To review the literature would highlight the novelty of the findings described in this manuscript.
4. The manuscript is very difficult to follow in its current shape. It would benefit from streamlining. In addition, the flow of logic should be tightened. As a suggestion, the structure could be outlined as follows: (i) identification and characterization of SFH8 (ii) cleavage of SFH8 (iii) filamentous versus punctate appearance of SFH8 full length, variants or fragments (iv) the case for LLPS or liquid to solid transitions (v) the role of LLPS in PIN polarity (including Fig. 2F, 2G), auxin distribution (Fig. 9) and plant development, including gravitropism (Figs 2G, S3A). The discussion should start with a recapitulation of the results. A graphical abstract would also help the reader.

Minor comments:

5. The figures are difficult to navigate. It would help if they could be structured in a grid-like manner and if the reader could move through them either from left to right or from top to bottom. Different annotations (as opposed to the same annotation in a different color) such as arrows, arrowheads or asterisks should be used to label structures and salient features consistently and clearly throughout the manuscript. The text font on the figures is too large, such that the labels are at times longer than the panels. Furthermore, the labels have too much information; promoters, for example, could be in the legend and the minimal relevant designator on the panel.
6. In Figure 2A the authors refer to the role of SFH8 in the “modulation of development” (an action subtitle; would a descriptive subtitle not be preferable?); here they should stay closer to their observations and describe more specifically the impact on root growth.
7. In Fig 2C the authors write “The depletion of sfh8 leads to a reduced meristem size”; it is not clear what the arrow heads point to, nor how meristem size was assessed.
8. In Fig 2D the authors write “The reduction of sfh8 meristem size is due to compromised cell divisions”. However, the authors have not monitored cell expansion as a function of distance from the QC/ i.e. exit from the meristem, which also influences meristem size. The conclusion could be turned around to “compromised cell division **contributes** to the reduction in meristem size”.
9. Fig 2G is all important as it shows an impact of KISC disruption via transient over expression of the KIN7.3 tail on gravitropism; this should be quantified as shown for other mutants in S3A Fig (circular plots showing moderate gravity perception defects of *sfh8, k135, rsw4*, the double *rsw4 sfh8*, and *k135 sfh8* mutants).
10. It would simplify the main figures considerably if the authors could focus on region 3 and relegate other regions of the root to the supporting information files.
11. The legends of the figures 3B, 4A, S1B, S1C, S2A, S3A, S6A, S9B, S10A state that experiments were replicated multiple times. However, the exact number of replicates is not indicated.
